# Therapeutic Perspectives of SIRT6 Regulation: Computational Analysis of Activation and Inhibition by Bioactive Molecules

**DOI:** 10.1002/jmr.70016

**Published:** 2025-12-08

**Authors:** Érika Geicianny de Carvalho Matias, Katyanna Sales Bezerra, Washington Sales Clemente Junior, Jonas Ivan Nobre Oliveira, Douglas Soares Galvão, Umberto Laino Fulco

**Affiliations:** ^1^ Departamento de Biofísica e Farmacologia Universidade Federal Do Rio Grande Do Norte Natal Brazil; ^2^ Applied Physics Department University of Campinas Campinas Brazil

**Keywords:** bioactive molecules, DFT, MFCC, Sirt6

## Abstract

Sirtuin 6 (SIRT6) is an enzyme belonging to the class of nicotinamide adenine dinucleotide (NAD+) dependent histone deacetylases. It has been of interest due to its multivariate biological role and association with aging‐related diseases and metabolic dysfunctions. SIRT6 activation protects against metabolic diseases and aging, and its inhibition is considered a therapy against cancer and inflammation. Here, we explore the modulation of SIRT6 by bioactive molecules, providing a detailed view of the molecular interactions that lead to the activation or inhibition of this protein. Therefore, we investigated the interactions between the ligands quercetin (QUE), isoquercetin (ISO), catechin gallate (CG), and trichostatin A (TSA) with SIRT6, using computational methods from the perspective of molecular modeling through the Molecular Fractionation with Caps Conjugates (MFCC) technique and according to the calculation parameters of Density Functional Theory (DFT). The results revealed the energetic values of each amino acid residue constituting the interaction pocket with the analyzed ligands within a radius of up to 10.0 Å. The analysis of the interaction energies showed an order of priority among the ligands, highlighting CG as the most promising. The observation of the interactions between amino acid residues and ligands identified significant contributions from residues VAL70, PHE64, PHE82, and PHE86. In addition, residues such as PRO62, MET136, MET157, and VAL115 stand out as key components of the protein active site. These findings offer strategic insights into the molecular mechanisms underlying the binding of the studied ligands to SIRT6, providing a deep understanding of their affinity and pharmacological potential.

## 1 Introduction

As metabolic diseases and cancer continue to pose significant public health challenges worldwide, affecting millions of individuals, it becomes imperative to delve into their complex etiology, which involves a nuanced interplay of genetic predisposition and environmental influences [1, 2, 3]. Metabolic disorders such as diabetes and obesity have garnered increased attention in recent decades due to shifting lifestyle patterns, with projections indicating a worrisome rise in their prevalence rates. This underscores the ongoing necessity for rigorous scientific inquiry to unravel the underlying mechanisms and develop effective treatment modalities. Within this landscape, the SIRT6 protein emerges as a focal point of interest owing to its pivotal role in regulating metabolic processes, cancer progression, and aging mechanisms [4].

Sirtuins constitute a broad family of nicotinamide adenine dinucleotide (NAD+) dependent enzymes that catalyze the deacetylation of proteins and are present in all domains of life, including archaea, eubacteria, yeast, plasmodia, metazoans, mammals, and even viruses [5, 6, 7, 8]. In mammals, these enzymes comprise seven homologs (SIRT1–SIRT7), which present variations in terms of tissue specificity, subcellular localization, enzymatic activity, and targets [6, 9]. Sirtuins act by deacetylating histones and a wide variety of proteins in different subcellular compartments [5, 10].

In general, these enzymes have a standard catalytic structural projection, which allows them to recognize NAD+ universally; however, the N and C terminal domains diverge and are possibly related to their respective biological, enzymatic functions, the binding of specific molecules, their subcellular localization, and expression patterns [11, 12]. Studies developed in recent decades have elucidated relevant information about the role that this family of proteins plays. They have been implicated in the regulation of energy metabolism in a variety of tissues and other fundamental biological functions, such as DNA repair, cell survival, response to stress, telomere and chromatin regulation, autophagy, cancer metabolism, gene silencing, apoptosis, learning, memory, sleep, circadian rhythm, and longevity [13, 14, 15, 16].

Among the mammalian Sirtuin family members, SIRT6 is an important regulator in several processes, including DNA repair, gene expression, telomere maintenance, metabolism, and aging. SIRT6 acts both through the deacylation of acetyl groups and long‐chain fatty acyl groups such as myristoyl, as well as a mono‐ADP‐ribosyl transferase [17, 18]. SIRT6 is a complex protein with additional enzymatic mechanisms and substrates beyond histone deacetylation. Furthermore, SIRT6 promotes the mono‐ADP‐ribosylation of specific factors involved in DNA repair and chromatin silencing, contributing to genomic stability. The protein also catalyzes the deacetylation of long‐chain acyl groups in proteins, modifications whose physiological functions are not yet fully elucidated. These multifaceted enzymatic activities position SIRT6 as a central regulator connecting chromatin dynamics to the regulation of cellular metabolism and stress responses [19].

**FIGURE 1:**
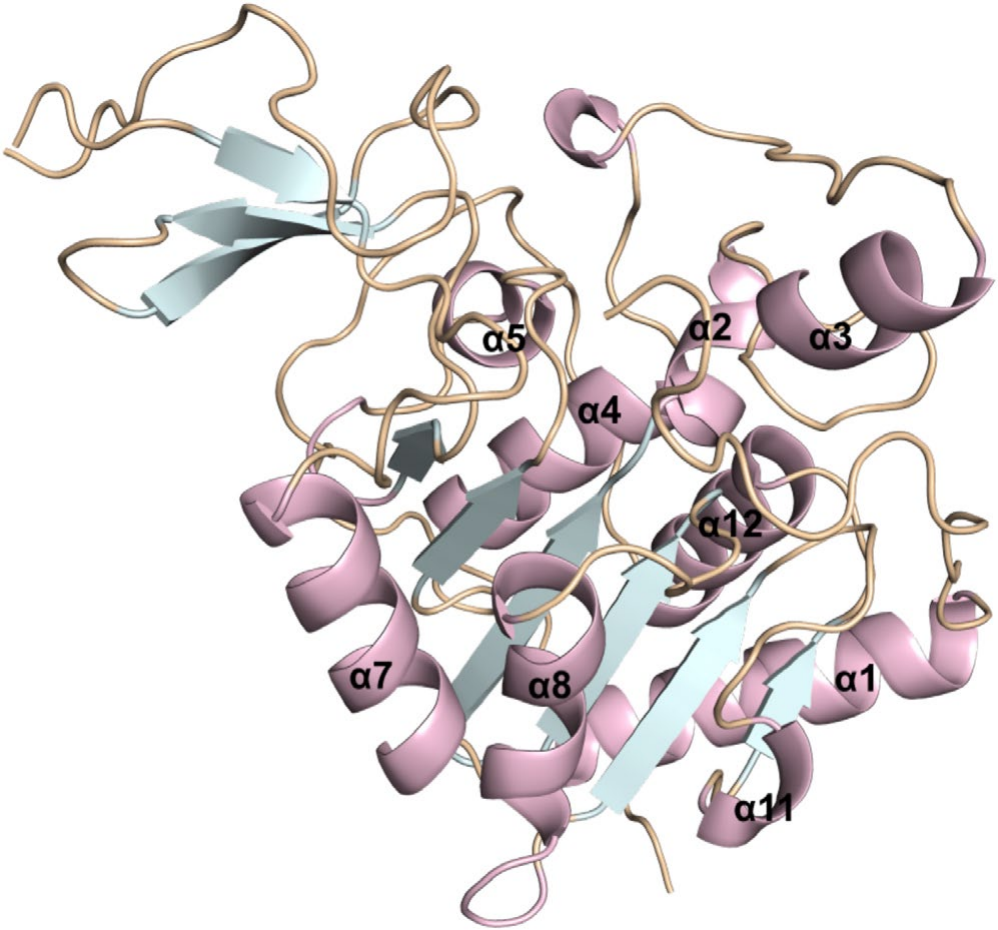
Representation of the structural characteristics of the SIRT6.

The protein structure of SIRT6 consists of a catalytic core formed by 275 amino acids, with a Rossman fold and a small Zn2+ binding domain. It presents a large binding pocket of hydrophobic nature in which there is a loop where the NAD+ cofactor is associated, as well as a channel for binding to the acyl group, favoring the accommodation of long‐chain acyls. Furthermore, its catalytic core is surrounded by N‐ and C‐terminal extensions, contributing to its association with chromatin [20]. SIRT6 features two globular domains, a large Rossmann fold, and a small zinc‐binding pocket consisting of eight α‐helices and nine β‐strands. The Rossmann fold domain is composed of six parallel β‐sheets (β 1, β 2, β 3, β 7, β 8 and β 9) located between two α‐helices (α 6 and α 7) on one side and four α‐helices (α 1, α 4, α 5 and α 8) on the other side. The zinc‐binding domain is composed of three antiparallel β‐sheets (β 4, β 5, and β 6) connected to the extension loops of two α‐helices (α 3 and α 6). As this protein is dependent on the NAD+ cofactor, its Rossmann fold offers two binding pockets, one for NAD+ and the other for the acyl substrate. The adenosine moiety of NAD+ is anchored at the site close to α 8 and β 8, and the nicotinamide moiety, NAM, is positioned in an open pocket around the N‐terminal loop [17, 21]. Figure 1 shows the structural characterization of SIRT6.

SIRT6 is recognized as a histone deacetylase (HDAC), with specific targets H3K9, H3K56, and H3K18, promoting the deacetylation of lysines at K9, K56, and K18, where this removal of the acetyl functional group of lysines appears to be fundamental for chromatin compaction repression of transcriptional and DNA damage responses. Thus, SIRT6 acts as a co‐repressor of several transcription factors involved in aging, cancer, and metabolism, allowing DNA damage‐dependent chromatin modifications that are essential for DNA repair and maintaining telomeric chromatin structure, preventing genomic instability and cellular senescence [22, 23].

Thus, modulation of SIRT6 activity through small molecules presents a promising avenue for advancing new therapeutic interventions, offering potential advances in addressing these complex health challenges with greater precision and efficacy in the coming years. Activation of SIRT6 protects against metabolic and aging‐related diseases, and its inhibition is considered a therapy against cancer and inflammation [24, 25, 26].

A key distinction between SIRT6 activators and inhibitors is related to their binding mode to this protein. Activators described in the literature, including natural flavonoids (such as quercetin, isoquercetin, and anthocyanidins) and synthetic compounds (e.g., MDL‐800, UBCS039), generally bind to allosteric sites distant from the catalytic site, promoting conformational changes that increase catalytic activity. In contrast, inhibitors such as trichostatin A interact preferentially with the active site or adjacent regions, competing directly with NAD+ or the peptide substrate, or even perturbing critical catalytic residues, such as His131, compromising deacetylase function. Furthermore, some flavonoids have demonstrated dual (activator/inhibitor) behavior, possibly due to their ability to interact simultaneously with allosteric and catalytic regions, which depends on concentration and experimental context [25, 26].

Flavonoids are among the molecules recognized as activators of SIRT6, and they show potential for treating metabolic, inflammatory, and anti‐cancer disorders. Molecules such as catechin gallate and trichostatin A are identified as potent SIRT6 inhibitors [27, 28, 29], while isoquercetin demonstrates activator activity for Sirt6 [30]. However, the flavonoid quercetin can function as both an activator and an inhibitor of SIRT6, depending on the concentration applied. In this context, these biomolecules can be used as a basis for the creation of selective and high‐potency structures targeting SIRT6 [31].

The study by Jos et al. [32] performed in silico screening for human SIRT6 modulators using a repository of deidentified patient‐level data from phase III clinical trials and molecular dynamics (MD) simulations to analyze protein‐ligand interactions and validate their binding affinity. Parenti et al. [33] presented, through computer screening, the identification of the first selective inhibitors of SIRT6; in this analysis, micromolar IC50 shows a significantly greater selectivity for SIRT6 compared to SIRT1 and SIRT2. Other research, using docking and molecular dynamics and in vitro analysis, demonstrated that a compound derived from imidazole offers a promising therapeutic strategy for the treatment of cancer, acting through the modulation of sirtuins and potentially interrupting key processes in tumor progression [34].

On the other hand, Rahnasto‐Rilla et al. [35] identified that flavonoids can alter the activity of SIRT6 in a structure‐ dependent manner. In this study, it was shown that the binding site for SIRT6‐activating molecules is found close to a loop close to the binding site for acetylated peptide substrates, suggesting that this moiety may play a role in SIRT6 activation. Although several studies evaluate the relevance of regulators for SIRT6 as a possible therapeutic approach, there are no investigations into the energetics of interaction between the protein and ligands using molecular partitioning methods. Molecular fragmentation methods have stood out in the field of computational studies, and recently, the Molecular Fragmentation with Conjugated Caps (MFCC) technique, where the total energy is considered as a sum of fractional energies, has provided plausible results for biomolecular systems. The MFCC has emerged as prominent due to its positive results [36, 37, 38, 39, 40].

Understanding the intermolecular interactions between SIRT6 and molecules with modulatory competence is crucial to advancing our understanding of the biological processes regulated by this enzyme and exploring its therapeutic potential. However, investigation of these modulators often involves time‐consuming and expensive experimental methods. In this context, this study consists of the use of quantum mechanics, specifically calculations within the Density Functional Theory (DFT) formalism, also using the MFCC fragmentation method as a promising and efficient approach to predict the interactions between SIRT6 and quercetin ligands (QUE), isoquercetin (ISO), catechin gallate (CG), and trichostatin A (TSA). By emphasizing the importance of quantum mechanical studies, this work aims to advance the understanding of SIRT6 modulators through the application of the MFCC‐based computational approach. Unlike conventional experimental assays, which are resource‐intensive, time‐consuming, and limited in resolving residue‐level interactions, or molecular dynamics simulations, which require substantial computational resources and limited simulation times, the MFCC strategy allows the calculation of interaction energies at the amino acid level while preserving the chemical context of the protein environment, in addition to mapping the contributions of all relevant residues and reducing the computational cost compared to calculations of complete quantum systems, thus providing an efficient alternative for analyzing protein‐ligand interactions in large biomolecular systems.

## 2 Materials and Methods

The investigations to calculate the interaction energies mentioned in this work were made possible using data from crystallographic structures obtained through X‐ray diffraction and available in the PDB repository (http://www.rcsb.org). The complexes examined in this study are composed of the ligands SIRT6, which were co‐crystallized with four different substances: quercetin (QUE), isoquercetin (ISO), catechin gallate (CG) and trichostatin A (TSA). The corresponding structures are designated by the codes (and their resolutions): 6QCD (1.84 Å), 6QCE (1.90 Å), 6QCJ (2.01 Å), and 6HOY (1.70 Å), respectively [26, 30]. Furthermore, amino acid side chains not determined by X‐ray diffraction were considered. To analyze the protonation state of the ligands and receptor amino acids of SIRT6 under physiological pH conditions, version 18.24 of the software Marvin Sketch (ChemAxon, https://www.chemaxon.com) in conjunction with the PROPKA 3.1 package [41].

Hydrogen atoms were introduced into the ligands, amino acids, and water molecules through protonation assessment. While the constituent atoms of the amino acid backbone were fixed, the remaining atoms present in the ligands, proteins, and water molecules were subjected to classical geometric optimization processes. Classical calculations were conducted using the CHARMM force field (Chemistry at Harvard Molecular Mechanics) [42], with the convergence criteria set at 10−5 kcal mol^−1^ (total energy change) and 10^−2^ kcal mol^−1^ Å^−1^ (RMS gradient).

Firstly, the protein was subdivided into its constituents, the amino acids, using the MFCC method, as described in Equation (1). This method fragments the protein structure into discrete amino acid units, breaking the corresponding peptide bonds. Recent contributions to quantum chemical fragmentation approaches have established MFCC as a functional approach for obtaining residue‐level interaction energies in large biomolecules. MFCC has been applied to several protein–ligand and protein–protein systems, demonstrating that it reproduces important interaction patterns while significantly reducing the cost of quantum system calculations [43, 44, 45, 46, 47, 48, 49, 50, 51, 52, 53, 54].

In order to preserve the local chemical context and meet valence requirements, each fragment is surrounded by a pair of conjugated caps. At the same time, hydrogen atoms are introduced into the molecular caps to prevent any unsaturated bonds [55]. Thus, the ligands are designated as L. In contrast, the amino acid residues interacting with these ligands are represented by R_i_, where *i* highlights the index of the *i*th amino acid residue. Each shell, C_i_ (C_i_ *), is made up of the adjacent residue covalently bonded to the amine (or carboxyl) group of residue R_i_, along the protein chain, allowing, thus, a more accurate description of the surrounding electronic environment [53]. Energy calculations for these fragmented structures were conducted based on density functional theory (DFT), allowing the determination of the interaction energy between the ligand and the individual fragment, IEM F CC (L − R_i_).

(1) $$\begin{array}{c} E\left(L-R_{i}\right)=E\left(L-C_{i-1}R_{i}C_{i+1}\right)-E\left(L-C_{i-1}C_{i+1}\right)\\ \;-E\left(C_{i-1}R_{i}C_{i+1}\right)+E\left(C_{i-1}C_{i+1}\right), \end{array}$$

In the equation, the first component, $E\left(L-C_{i-1}R_{i}C_{i+1}\right)$, represents the total energy of the system involving the ligand and the residue under analysis and its caps; the second term, $E\left(L-C_{i-1}C_{i+1}\right)$, indicates the global energy of the capped residue; the third component, $E\left(C_{i-1}R_{i}C_{i+1}\right)$, describes the total energy associated with the caps and the ligand; finally, the fourth element, $E\left(C_{i-1}C_{i+1}\right)$, expresses the energy of the caps with the free bonds neutralized by hydrogens. Furthermore, in the methodology adopted, the water molecules present in the crystallographic structure were meticulously included in the computational calculations, being bonded to a residue Ri or to one of its ends when an H‐bond is established between them. In the case of multiple hydrogen interactions between a water molecule and several amino acids, the amino acid with the closest bond to water was prioritized to establish the bond.

To analyze the structures resulting from the fragmentation of SIRT6‐ligand complexes, computational energy calculations were performed using the Gaussian (G09) [56] software, based on density functional theory (DFT). The quantum in silico approach was performed with the B97D generalized gradient approximation (GGA) functional [57]. In order to describe the wave function of electrons and expand the Kohn‐Sham orbitals covering all electrons, we chose to employ the set 6‐311+G(d,p). This basis set includes triple zeta valence functions, additional polarization functions (d,p), and a fuzzy function (+).

It is important to note that choosing different functionals and basis sets can affect the calculated relative binding energy. However, in a study conducted by Barbosa and collaborators [58], nine different groupings were tested between functionals and base sets to calculate the interaction energy between the zinc ion and the residues in the metal binding site of the porphobilinogen synthase. It was observed that such variations do not affect the identification of the most relevant amino acid residues for the evaluated system, which is the main focus of our investigation. The choice of functional is also supported by the analysis of an extensive range of data sets, according to the study proposed by Li et al., where B97D stood out as the best performer compared to some of the hybrid methods used. Furthermore, it demonstrated an efficiency comparable to functionals incorporating D3 corrections [59].

Ensuring an accurate representation of the electrostatic environment is a crucial step in theoretical studies of biomolecular properties. Although proteins generally have a dielectric constant of around 4, in an aqueous solvent, this constant reaches 78; previous studies that used the MFCC method explored different values for this constant. It was found that a value of ε = 40 presented a satisfactory correspondence between simulations and relevant experiments [60, 61]. Consequently, we employed the Continuous Polarizable Conductor Method (CPCM) [62], setting the dielectric constant (*ε*) at 10 and 40 to characterize the environment adjacent to each segment obtained through the MFCC method. Additionally, according to research conducted by Vicatos et al. [63] adjusted the dielectric constant in a model designed to predict the stability of proteins and concluded that the same value (*ε* = 40) provided the best fit, improving consistency between calculations and observations. Similarly, Morais et al. [64], who contrasted homogeneous and heterogeneous dielectric models, highlighted that a high dielectric constant, around 40, represents an appropriate choice to describe the electrostatic environment at the interface between protein complexes.

Aiming to preserve the essential interactions, an analysis of the stability of the total binding energy was carried out in relation to the radius r of the ligand binding cavity, limiting the number of amino acid residues to be considered. For this, imaginary spheres were established (with *r* = *n*/2; *n* = 1, 2, 3, 4, …), outlining increasing distances from the ligands to calculate the sum of the individual energies of the residues present in each sphere. Thus, stability is achieved when the energy variation in the subsequent beam is less than 10% [65].

## 3 Results and Discussion

### 3.1 Convergence Analysis

**FIGURE 2:**
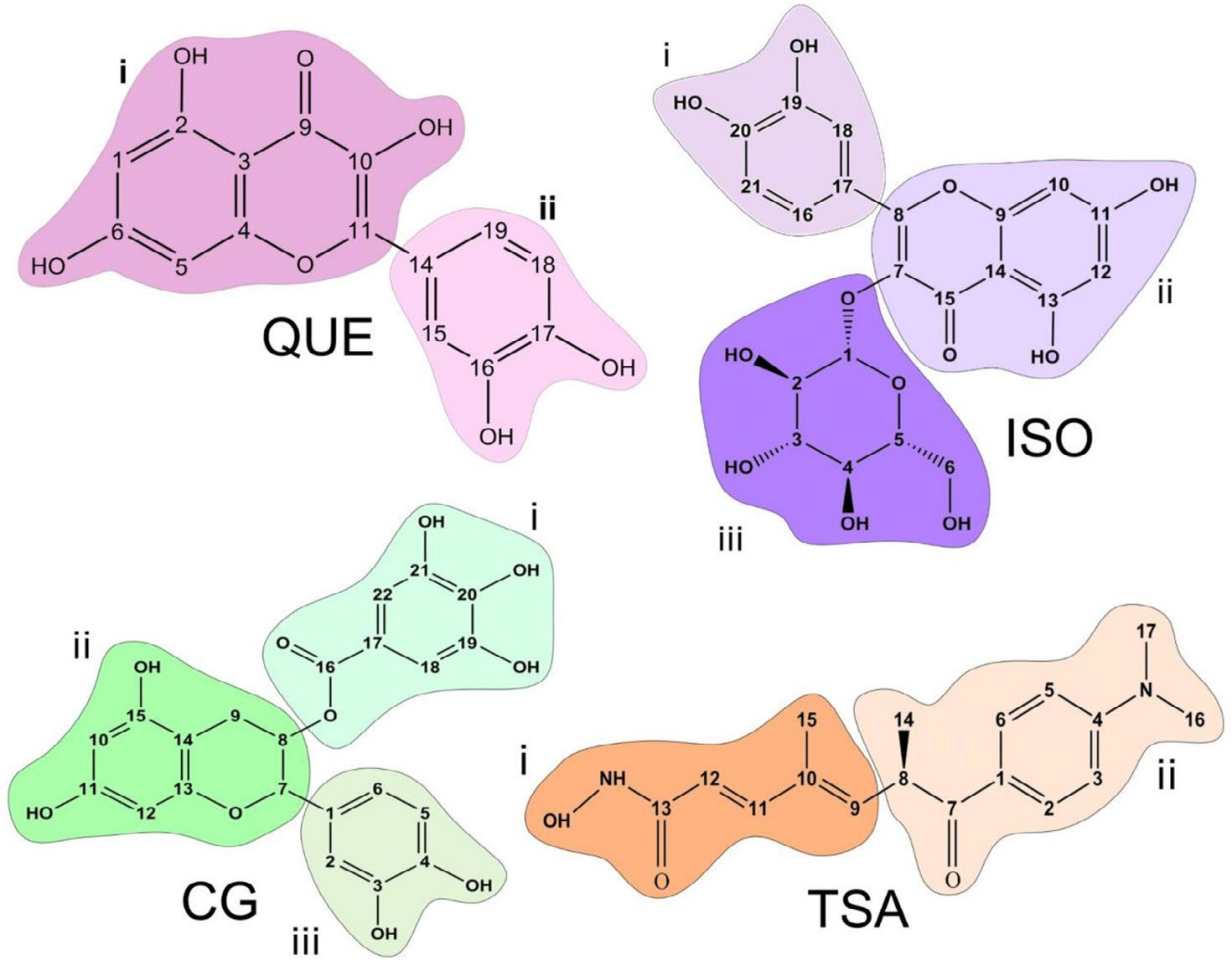
Illustrative visualization of the chemical structures of the ligands, namely QUE (Quercetin), ISO (Isoquercetin), CG (Catechin galate), and TSA (Trichostatin A), subdivided into regions colored pink, purple, green, and orange, respectively.

To describe the interactions of each ligand with the SIRT6 protein in more detail, we divided the four ligands into specific regions for a clearer presentation and discussion of the results (see Figure 2). Therefore, Quercetin (QUE) and Trichostantin A (TSA) ligands were subdivided into two regions (i, ii), while Isoquercetin (ISO) and Catechin gallate (CG) were divided into three regions (i, ii, iii).

The energetic interactions and convergence criteria were examined for the four different ligands, each determined by their respective distance r (in units of Å) and the corresponding interaction energies (in kcal/mol). To evaluate the interactions between the SIRT6‐ligand complexes, all amino acids with attractive or repulsive potential that could significantly influence the system were considered. The total binding energy for each interaction radius was calculated by summing the individual contributions of the amino acids for which convergence was achieved. When considering the energy of attractive (negative energy) or repulsive (positive energy) interactions for each relevant amino acid, the analysis highlighted the optimal radius r of the binding site, which does not substantially alter (less than 10%) the energy of total binding.

**FIGURE 3:**
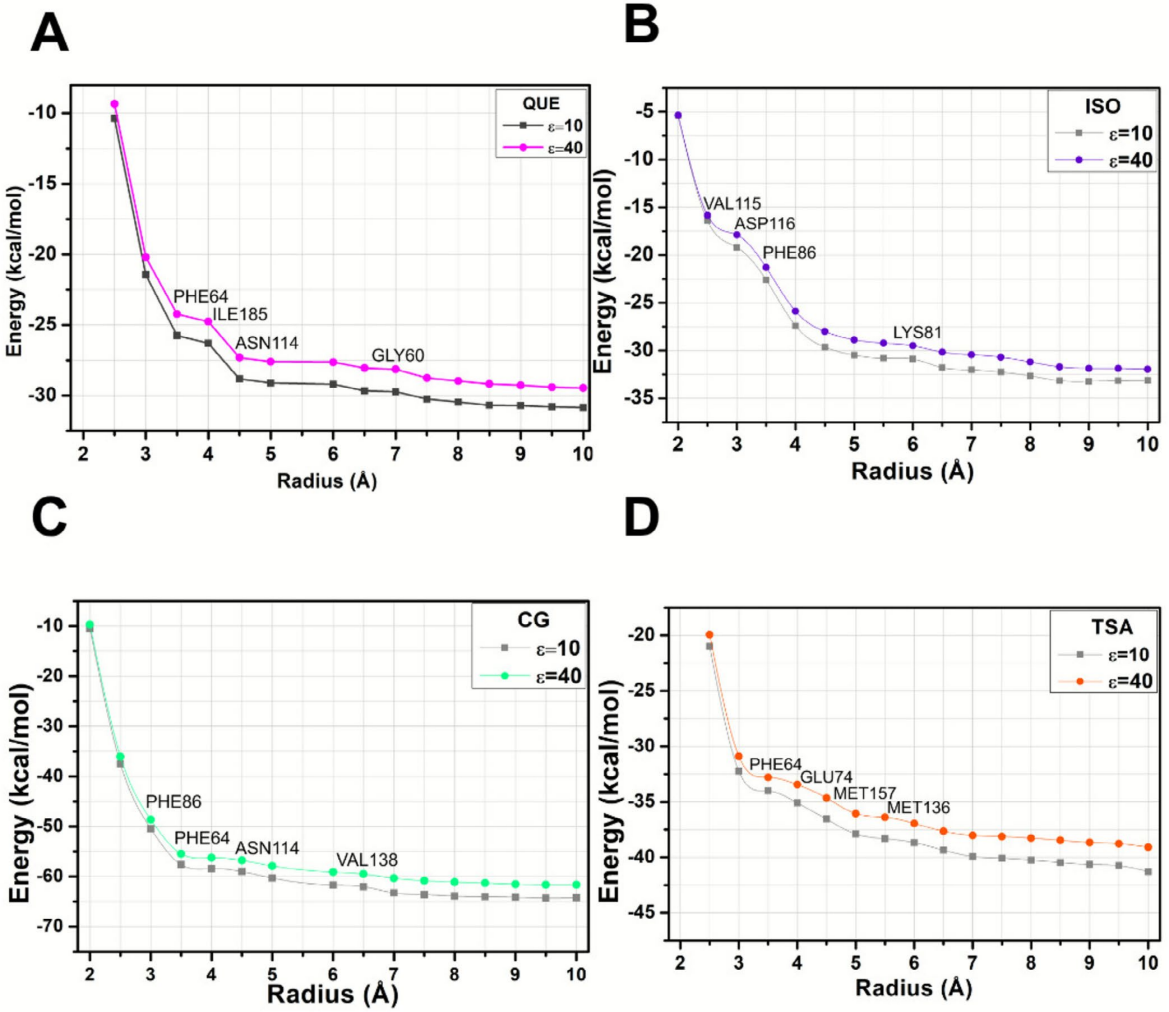
Illustration depicting the general interaction energy of the complexes SIRT6‐QUE (A), SIRT6‐ISO (B), SIRT6‐CG (C), and SIRT6‐TSA (D) in relation to the radius of the binding pocket r, calculated using the GGA B97D functional under the MFCC structure and employing two dielectric constants (*ε* = 10, *ε* = 40).

The values of the total binding energies calculated for the interaction of the SIRT6‐ligand complexes as a function of the values of the radius *r*, and considering the dielectric constants *ε* = 10 and 40, are presented in Figure 3A–D. It is observed that convergence was achieved for radii *r* = 6.0, 5.5, 7.0, and 6.0 Å (assuming ε = 40) for QUE, ISO, CG, and TSA, respectively. However, to account for the influence of a substantial amount of residue and ensure that all relevant contributions to interactions between systems are considered, we conducted all analyzes up to a radius *r* = 10.0 Å, resulting in a total of 66 (QUE), 83 (ISO), 81 (CG), and 84 (TSA) interactions identified. Interaction energies were calculated up to a radius of 10.0 Å to ensure that all relevant amino acid residues contributing to ligand binding were comprehensively considered. This ensured the inclusion of the full extent of the binding site environment, confirming that no significant interactions beyond this distance were missed. The choice of a 10 Å cutoff is consistent with previous computational studies of protein‐ligand interactions, in which similar radii were used to encompass all residues that significantly contribute to binding energetics [47, 66].

### 3.2 Intermolecular Interactions of the SIR6 Complex‐Modulators

Analysis of the total interaction energy at the SIRT6 binding site for the compounds investigated in this study revealed the following values (*ε* = 10 and *ε* = 40): for quercetin (QUE), −30.85 and −29.46 kcal/mol; for isoquercetin (ISO), −33.12 and −31.95 kcal/mol; for catechin gallate (CG), −64.22 and −61.63 kcal/mol; and for trichostatin A (TSA), −41.29 and −39.08 kcal/mol. These results show a clear hierarchy of ligand affinities, where we observed the highest value for the interaction energy for CG, indicating a strong affinity for the binding site. TSA and ISO followed this, and finally, QUE exhibited the lowest interaction energy values, indicating a relatively lower affinity with the SIRT6 binding site compared to the other compounds studied.

By observing the graphs that indicate the convergence for the calculations of the four complexes, we can deduce that the energetic performance is satisfactorily equivalent for *ε* = 10 and *ε* = 40. Therefore, the results presented below are associated with the values acquired for ε40. All interaction energy calculations performed between SIRT6 and ligands are accessible in Tables S1, S2, S3, and S4 (see Supporting Information S1).

**FIGURE 4:**
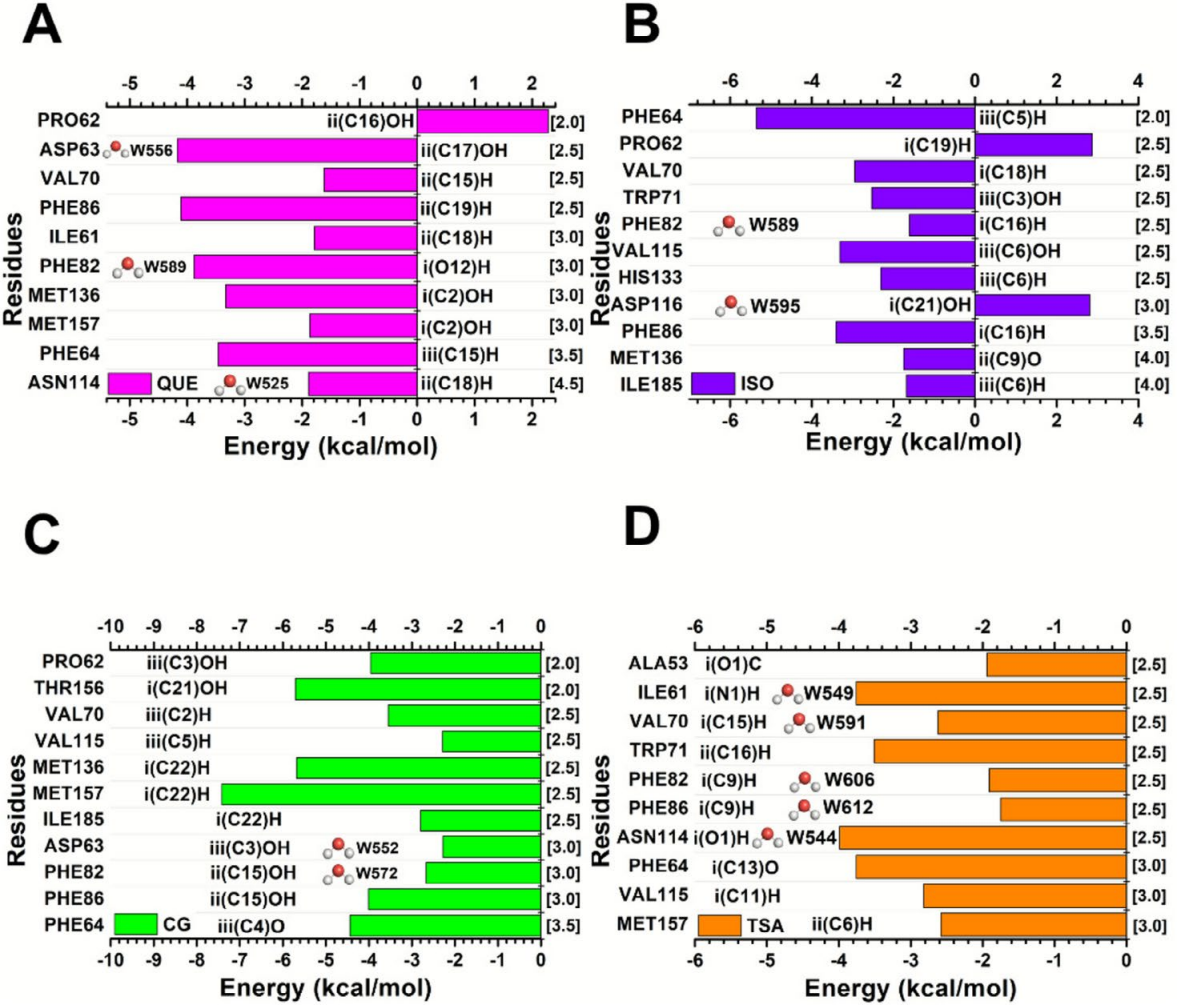
Graphic panels showing the most relevant residues regarding the contribution of the interaction energies of the three evaluated complexes: (A) SIRT6‐QUE (in pink), (B) SIRT6‐ISO (in purple), (C) SIRT6‐CG (in green), and (D) SIRT6‐TSA (in orange). Also shown are the region and atoms of the ligands that interact with each residue at the binding site and the interaction radius at which the residue is found. In addition, water molecules that can interact with the highlighted residues are also represented.

The context of the residues that make up the protein binding site determines the feasible interactions that encourage ligand binding. Therefore, characterizing the impact of these amino acid residues on the binding cavity is essential to gain an understanding of the functional diversity of ligands. Figure 4 shows the graphical schemes, including the interaction energies between the ligands QUE (Figure 4A), ISO (Figure 4B), CG (Figure 4C), and TSA (Figure 4D), and the amino acids with the most significant interaction energy for each complex measured. The graphs provide visual representations of the interaction energy (in kcal/mol) of the SIRT6 residue with a specific ligand, illustrated by the horizontal bars, demonstrating the quantitative values for *ε*40; on the left side of the graphs, the most important residues that contribute to the binding of the complexes the regions and atoms of the ligands in proximity to each residue in the binding site are indicated, while, on the right side, the interaction radii in which the amino acids are noted (in Å). In addition, water molecules that can associate with the highlighted amino acids are also displayed.

Based on the results of the intermolecular interactions between the SIRT6 protein and the investigated compounds, we highlight the most relevant interactions regarding interaction energy. The main intermolecular interactions observed between the SIRT6 protein and the modulators QUE, ISO, CG, and TSA are described below, highlighting the residues involved and the types of bonds established.

#### 3.2.1 SIRT6 in Complex With Quercetin (QUE)

The SIRT6–QUE binding site had 10 residues that were identified as highly influential. Among these, the amino acid PRO62 exhibited a repulsive interaction, as evidenced by a positive energy value (2.28 kcal/mol). In contrast, nine residues displayed attractive energies, reflected by negative energy values. Specifically, the most favorable interactions were observed with the following residues (in kcal/mol): ASP63 (–4.17), PHE86 (–4.11), PHE82 (–3.88), PHE64 (–3.46), MET136 (–3.33), MET157 (–1.87), ASN114 (–1.89), ILE61 (–1.79), and VAL70 (–1.62).

PRO62 appears to be a key amino acid, as the catechol portion of quercetin (ring B) is buried within the protein's binding cavity, and its hydroxyl group forms a hydrogen bond with the backbone oxygen of this residue [26]. However, a modification in the positioning of the nitrogen element in the aliphatic side chain of proline may reduce the affinity and activation of SIRT6 [25]. For this complex, PRO62 was characterized as relevant, interacting with QUE [ii(C16)OH] through hydrogen bonding. Nevertheless, this residue exhibited significant repulsive values at a 2.0 Å radius. This repulsion can be explained by the high electron density in the region near the diphenolic ring, due to hydroxyl (—OH) groups that interact with the specific electronic density of the PRO62 carbonyl group. Additionally, the rigid cyclic conformation of proline hinders the approach of the bulky flavonoid, contributing to the observed repulsion (see Figure 9A).

ASP63, PHE86, PHE82, and PHE64 exhibited the most significant attractive interaction energies. ASP63 interacted with QUE [ii(C17)OH] through a non‐conventional hydrogen bond. A mutation in this residue, D63H, results in the near‐complete loss of histone deacetylase function and is associated with human perinatal lethality, suggesting a crucial role of SIRT6 deacetylase activity in embryogenesis [67].

PHE86, PHE82, and PHE64 play an essential role in the catalytic channel or active site (site C), which is predominantly hydrophobic, where compounds such as QUE and other SIRT6 modulators bind [26, 31]. The amino acid PHE86 interacts with QUE [ii(C19)H] through dipole‐induced interactions, and π–π T‐shaped stacking interactions were also observed. PHE82 exhibited non‐conventional hydrogen bonding with QUE [i(O12)H], as well as T‐shaped π–π stacking networks. For PHE64, interaction occurred in the QUE [ii(C15)H] region via dipole‐induced bonding and π‐stacking. Molecular docking studies indicate that π interactions involving residues PHE86, PHE82, and PHE64 play an important role in the thermal stability of SIRT6, which, in certain cellular contexts, has been associated with inhibition of the PI3K/Akt signaling pathway in mouse embryonic fibroblasts [68].

Methionine residues MET136 and MET157 were also relevant in this complex. MET136 demonstrated attractive energetic interactions through non‐conventional hydrogen bonding with QUE [i(C2)OH]. MET157 showed significant attractive interactions with QUE [i(C2)OH] through electrostatic bonding. This occurred due to the polarization of the QUE hydroxyl group, allowing attraction between the partial positive charge of hydrogen and the partial negative charge of the methionine sulfur atom. In the same region, a conventional hydrogen bond and a π‐sulfur interaction were also observed. Previous studies have shown that these methionines form contacts with the quercetin rings (benzene ring A and pyran heterocyclic ring C), and the absence of a carbonyl group in this interaction confers greater potency, enabling optimal hydrophobic interaction between ring C and MET136/157. MET157 appears to be a key residue for enhancing potency within the binding site [25, 26].

**FIGURE 5:**
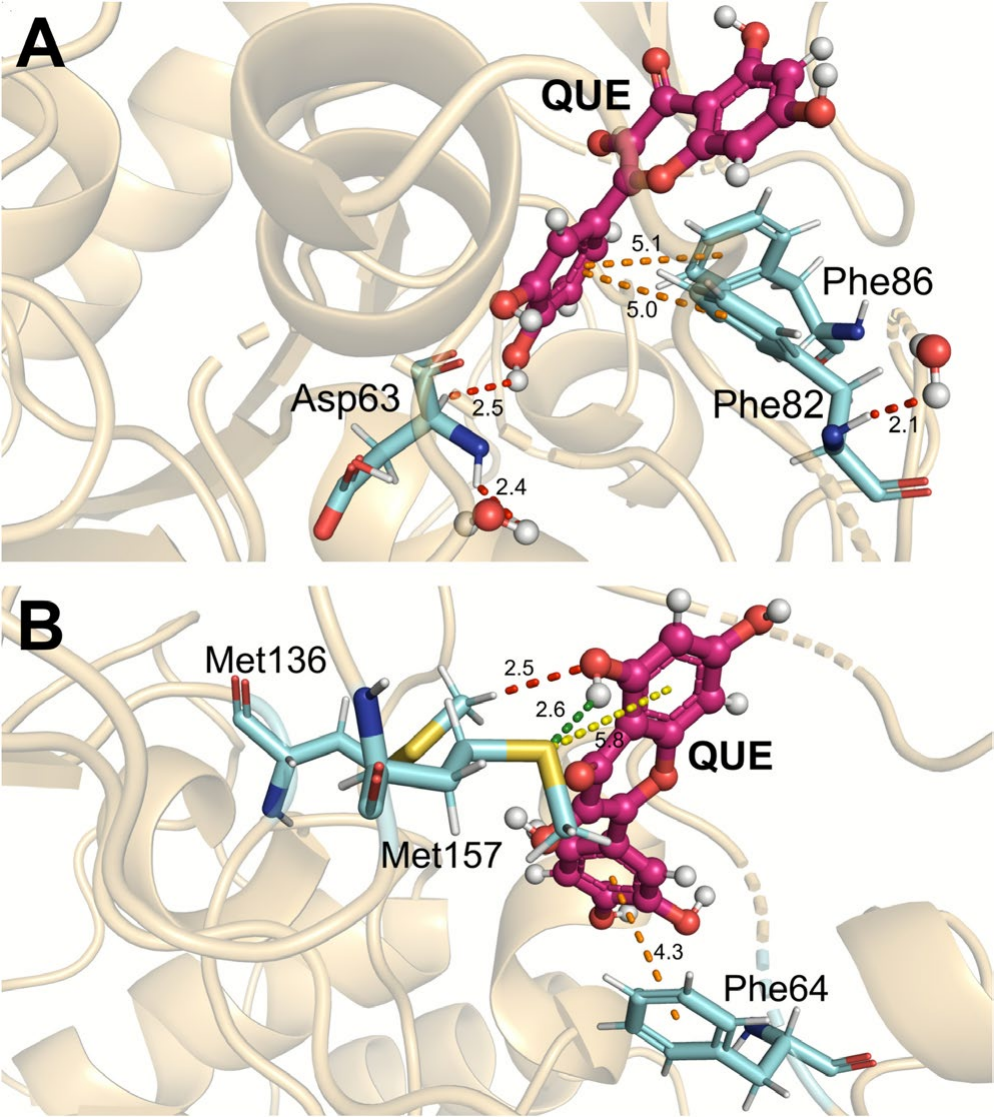
Schematic representation of interactions with key residues for the SIRT6‐QUE complex. (A) Intermolecular interactions between the QUE ligand and amino acids Asp63, Phe82, Phe86, and (B) with amino acids Phe64, Met136, and Met157. Dashed lines refer to non‐conventional H‐bonds (red), π‐alkyl (pink), electrostatic (green), π‐T‐Shaped/Stacked (orange), and π‐alkyl (yellow).

ASN114, ILE61, and VAL70 also exhibited significant energetic interactions. The amino acid ASN114 interacted with QUE [ii(C18)H] through non‐conventional hydrogen bonding. This amino acid is primarily located at the base of the hydrophobic channel of SIRT6, where it can form polar interactions with ligands [47]. On the other hand, ILE61 and VAL70 exhibited dipole‐induced interactions with QUE [ii(C18)H]/[ii(C15)H]; both residues constitute part of the hydrophobic pocket of SIRT6, which is essential for ligand binding [25, 69]. Figure 5A,B represent the main intermolecular interactions between SIRT6 and the QUE.

#### 3.2.2 SIRT6 in Complex With Isoquercetin (ISO)

For the SIRT6–ISO complex, 11 amino acids were identified as relevant. Among them, two residues exhibited repulsive interactions: PRO62 (2.87 kcal/mol) and ASP116 (2.81 kcal/mol). The remaining nine residues showed attractive interactions, as follows (in kcal/mol): PHE64 (–5.36), PHE86 (–3.41), VAL115 (–3.31), VAL70 (–2.95), TRP71 (–2.53), HIS133 (–2.31), MET136 (–1.75), ILE185 (–1.68), and PHE82 (–1.61).

The residues PRO62 and ASP116 exhibited significant, yet repulsive, interactions with the activator ISO. The repulsions occurred at radii of 2.5 Å and 3.0 Å, respectively. The repulsion involving PRO62 can be explained similarly to that observed in the QUE complex, while the repulsive interaction with ASP116 results from the proximity of negative charges between the carboxylic group of aspartic acid and the diphenolic ring of ISO. These repulsive forces may influence the ligand's orientation within the binding site, modulating SIRT6 affinity and activation potential (see Figure 9B,C).

PHE64, PHE86, VAL115, and VAL70 exhibited the most significant attractive interaction energies. PHE64 interacted with the activator ISO [iii(C5)H] through dipole‐induced intermolecular forces, also showing π–π stacked interactions. Similarly, PHE86 formed dipole‐induced interactions with ISO [i(C16)H]. Both phenylalanines (64 and 86) are located within the hydrophobic channel of SIRT6 and are important for ligand binding. Mutagenesis analyses revealed that Phe86, rather than Phe64 or Phe82, is primarily responsible for the π‐stacking effect. The Phe86 mutation, whose significance has been experimentally confirmed, resulted in reduced potency of the tested compounds (SIRT6‐F86A) [25, 70].

Valine residues VAL115 and VAL70 interacted with the activator ISO via dipole‐induced bonding at regions [i(C18)H] and [iii(C6)OH], respectively. The first binds to ISO through the adjacent, more robust portion of the glycosidic group [26]. The latter belongs to a distal portion of the acyl‐binding channel. This region is defined by the N‐terminal residues of SIRT6—Val70, Glu74, Phe82, Phe86, Val153, and Met157—and represents a hydrophobic pocket crucial for ligand interaction [69].

The amino acids TRP71, HIS133, and MET136 exhibited relevant interaction energies within the SIRT6–ISO complex. TRP71 interacted with ISO through non‐conventional hydrogen bonding. This residue was previously identified as one of those forming hydrophobic interactions with a potent synthetic activator of SIRT6, UBCS039 [25]. The interaction with HIS133 occurred through a dipole‐induced bond in the [iii(C6)H] region. This amino acid has also been reported as significant for interaction with another biomolecule, catechin gallate, which exhibits inhibitory activity against SIRT6 [30].

MET136 interacted with ISO via the [ii(C9)O] region through non‐conventional hydrogen bonding. Previous studies have shown that methionine MET136 forms contacts with the quercetin rings—benzene ring A and the pyran heterocyclic ring C. Since isoquercitrin is a derivative of quercetin, containing the same aromatic ring structure, these hydrophobic interactions are also expected to occur with ISO [25, 26].

**FIGURE 6:**
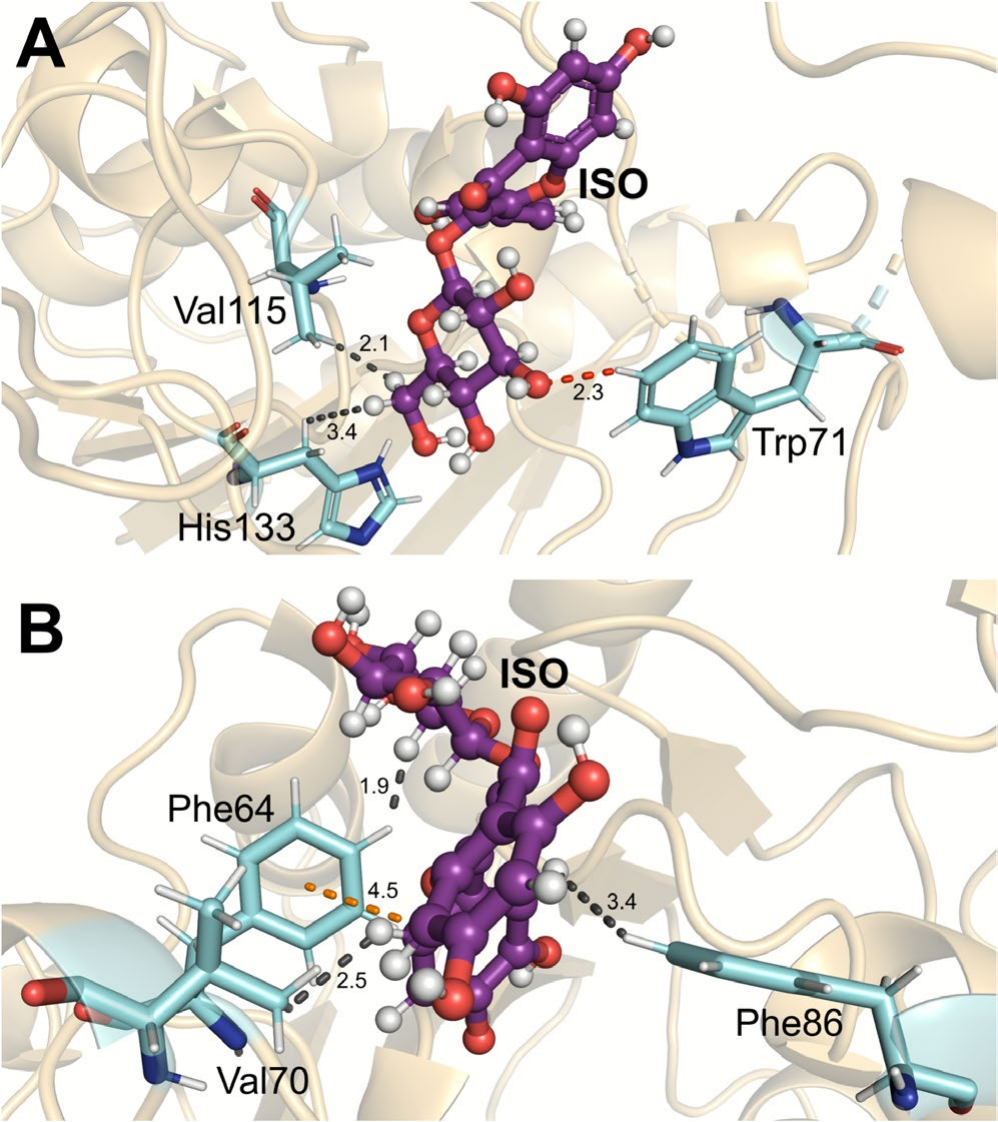
Schematic representation of the interactions with key residues for the SIRT6‐ISO complex. (A) Intermolecular interactions between the ISO ligand and amino acids Val115, His133, Trp71, and (B) with amino acids Phe64, Val70, and Phe86. Dashed lines refer to π‐T‐Shaped/Staked (orange) and dipole–dipole (black).

ILE185 and PHE82 were also significant for this complex. Both residues played an important role in ISO binding, interacting through regions [iii(C6)H] and [i(C16)H], respectively, via dipole‐induced interactions. A previous study observed that Ile185, together with residues Trp71, Phe82, Phe86, and Met157, displays a weakly hydrophobic character when interacting with a compound derived from UBCS039 [25]. Figure 6A,B represent the main intermolecular interactions between SIRT6 and the ISO.

#### 3.2.3 SIRT6 in Complex With Catechin Gallate (CG)

Regarding the interactions in the SIRT6–CG complex, 11 residues exhibited significant interaction energies, all of which were notably attractive. The corresponding values (in kcal/mol) are as follows: MET157 (–7.42), THR156 (–5.71), MET136 (–5.68), PHE64 (–4.44), PRO62 (–3.96), PHE86 (–4.01), VAL70 (–3.55), ILE185 (–2.80), PHE82 (–2.67), ASP63 (–2.28), and VAL115 (–2.29).

The amino acids MET157, THR156, MET136, and PHE64 exhibited the most energetically favorable interactions in this complex. MET157 interacted with CG [i(C22)H] through dipole‐induced bonds and π‐alkyl interactions. Similarly, MET136 displayed dipole‐induced bonding and π‐alkyl interactions with CG [i(C22)H]. Both methionines, MET157 and MET136, appear to be key residues that enhance binding‐site potency [25, 26].

The interaction between THR156 and the CG ligand [i(C21)OH] occurred through hydrogen bonding. This amino acid has been described as an important residue within the SIRT6 active site for binding quercetin and its derivative cyanidin, interacting with the catechol portion of these flavonoids through hydrogen bonds involving its backbone [25].

For PHE64, hydrogen bonding was observed with CG [iii(C4)O], in addition to π‐alkyl interactions. Molecular docking studies have revealed that π interactions involving residues Phe64, Phe82, and Phe86 play a significant role in the thermal stability of SIRT6. In certain cellular contexts, Phe64‐mediated interactions have been particularly associated with inhibition of the PI3K/Akt signaling pathway in mouse embryonic fibroblasts [70].

The residues PRO62, PHE86, VAL70, and ILE185 also displayed notable interaction energies. PRO62 interacted with CG via hydrogen bonding at the [iii(C3)OH] region. The interaction with PHE86 occurred through non‐conventional hydrogen bonds at the [ii(C15)OH] region of CG. Phe86 is located in the hydrophobic channel of SIRT6 and is essential for ligand binding within the site. Mutational analyses revealed that this amino acid provides the primary π‐stacking effect [25, 67].

VAL70 and ILE185 interacted with CG through dipole‐induced interactions at [iii(C2)H] and [i(C22)H], respectively. Additionally, VAL70 exhibited π–alkyl interactions. Val70 belongs to a distal portion of the acyl‐binding channel, defined by the N‐terminal residues of SIRT6, representing a hydrophobic region important for ligand interaction [47]. A previous study also observed that Ile185 exhibits a weakly hydrophobic character in its interaction with a compound derived from UBCS039 [25].

**FIGURE 7:**
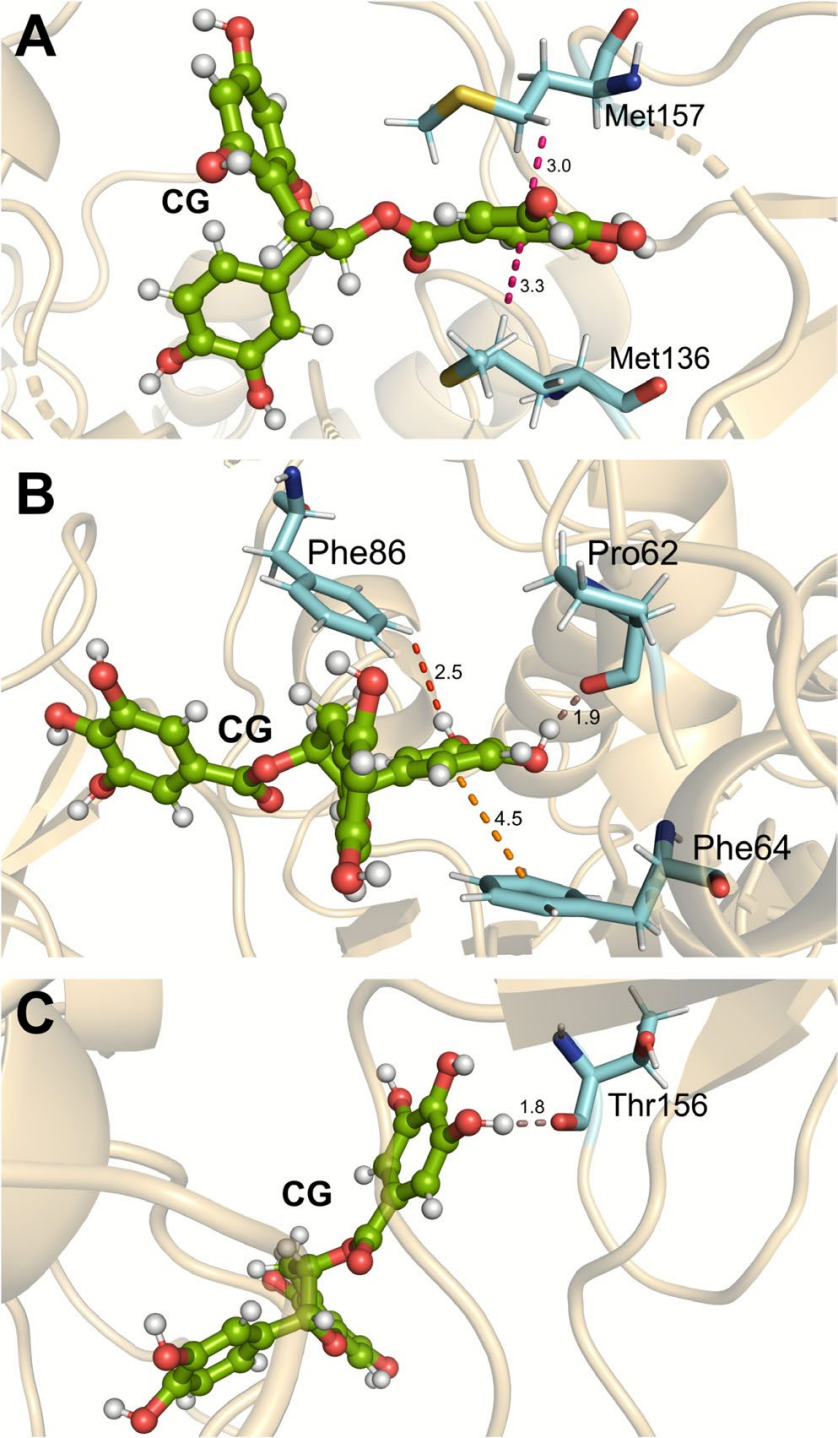
Schematic representation of the interactions with key residues for the SIRT6‐CG complex. (A) Intermolecular interactions between the CG ligand and amino acids Met136, Met157 (B) with amino acids Pro62, Phe64, Phe86, and (C) with residue Thr156. Dashed lines refer to H‐bonds (salmon), π‐alkyl (pink), and dipole–dipole (black).

The amino acids PHE82, ASP63, and VAL115 were also significant in this complex. PHE82 interacted with CG via non‐conventional hydrogen bonding at the [ii(C15)OH] region, as well as through π interactions. This residue is part of the hydrophobic channel of SIRT6 and contributes to ligand binding within the site [25, 68]. ASP63 exhibited non‐conventional hydrogen bonding with CG [iii(C3)OH]. An in vitro study demonstrated that the D63H mutation results in an almost complete loss of histone deacetylase activity, which is associated with perinatal lethality in humans. These findings suggest that the deacetylase function of SIRT6 plays an essential role during embryogenesis [69]. Finally, the interaction with VAL115 occurred at the CG [iii(C5)H] region through dipole‐induced bonding. Figure 7A–C represents the main intermolecular interactions between SIRT6 and the CG.

#### 3.2.4 SIRT6 in Complex With Trichostatin A (TSA)

Similarly, for the SIRT6‐TSA complex, 10 residues were identified as important, namely (in kcal/mol): ASN114 (−3.99), PHE64 (−3.76), ILE61 (−3.76), TRP71 (−3.51), VAL115 (−2.82), VAL70 (−2.62), MET157 (−2.58), ALA53 (−1.94), PHE82 (−1.91), and PHE86 (−1.75).

The amino acids ASN114, PHE64, ILE61, and TRP71 were the most energetically significant. ASN114 interacted with TSA [i(O1)H], showing a hydrogen bond. ASN114, as well as Ser56 and Pro62, are primarily located at the base of SIRT6's hydrophobic channel; these residues can form polar interactions with ligands [63]. The interaction of PHE64 occurred through a hydrogen bond in the TSA region [i(C13)O]. Studies indicate that this residue plays a relevant role in SIRT6's thermal stability [62]. ILE61 exhibited dipole‐induced interactions with TSA [i(N1)H]. Ile61 is part of the hydrophobic pocket also formed by Phe64, Phe82, Phe86, Pro62, and Met136, which are important for ligand binding [25].

TRP71 was significant for TSA compounds [ii(C16)H], showing T‐shaped π‐π stacking interactions. The interaction of this residue with the TSA modulator occurs with the hydroxamate portion buried in the active binding site of SIRT6 (site C), mimicking the interactions performed by the NAM portion of NAD+ [26]. TRP71 was also identified as one of the residues forming hydrophobic interactions with a potent synthetic SIRT6 activator, UBCS039 [25].

VAL115, VAL70, and MET157 were important for this complex. These amino acids interacted with TSA through dipole‐induced bonds via the regions [i(C11)H], [iii(C2)H], and [ii(C6)H], respectively. Additionally, VAL70 and MET157 exhibited π‐alkyl interactions. The TSA modulator associates with the phenyl ring of the ligand oriented toward the exit of the channel [26]. Val70 and Met157 belong to a distal portion of the acyl‐binding channel. The hydrophobic region of SIRT6 is significant for interaction with ligands because of the N‐terminal residues [63].

ALA53 interacted with TSA via the [i(O1)C] region through dipole‐induced bonds. This amino acid has been shown to be a significant residue in the SIRT6 active site for the binding of quercetin and its derivative cyanidin, interacting with the catechol portion of these flavonoids through hydrogen bonds with the amino acid backbone [69].

**FIGURE 8:**
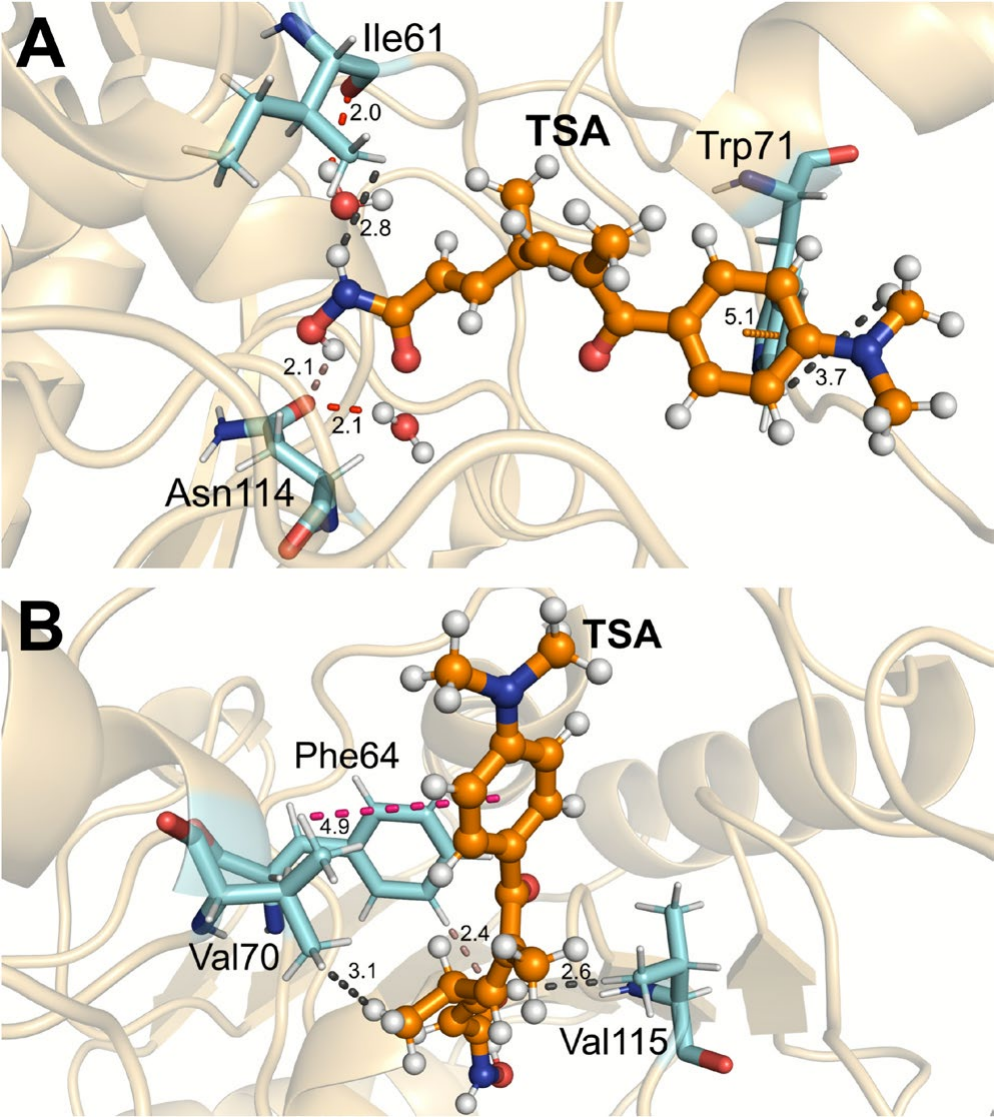
Schematic representation of the interactions with key residues for the SIRT6‐TSA complex. (A) Intermolecular interactions between the TSA ligand and amino acids Trp71, Ile61, Asn114, and (B) with amino acids Val70, Val115, and Phe64. Dashed lines refer to H‐bonds (salmon), π‐alkyl (pink), dipole–dipole (black), and π‐T/Staked (orange).

Finally, the phenylalanines PHE82 and PHE86 interact with the TSA modulator through dipole‐induced bonds in the [i(C9)H] region, and π‐alkyl interactions were also identified with PHE82. Phe82 and Phe86 are in the hydrophobic channel of SIRT6 and are important for ligand binding to the site. Mutational analyses revealed that Phe86 and Phe82 provide the main π‐stacking effect [25, 70]. Figure 8A,B represents the main intermolecular interactions between SIRT6 and TSA.

### 3.3 Repulsive Interactions

**FIGURE 9:**
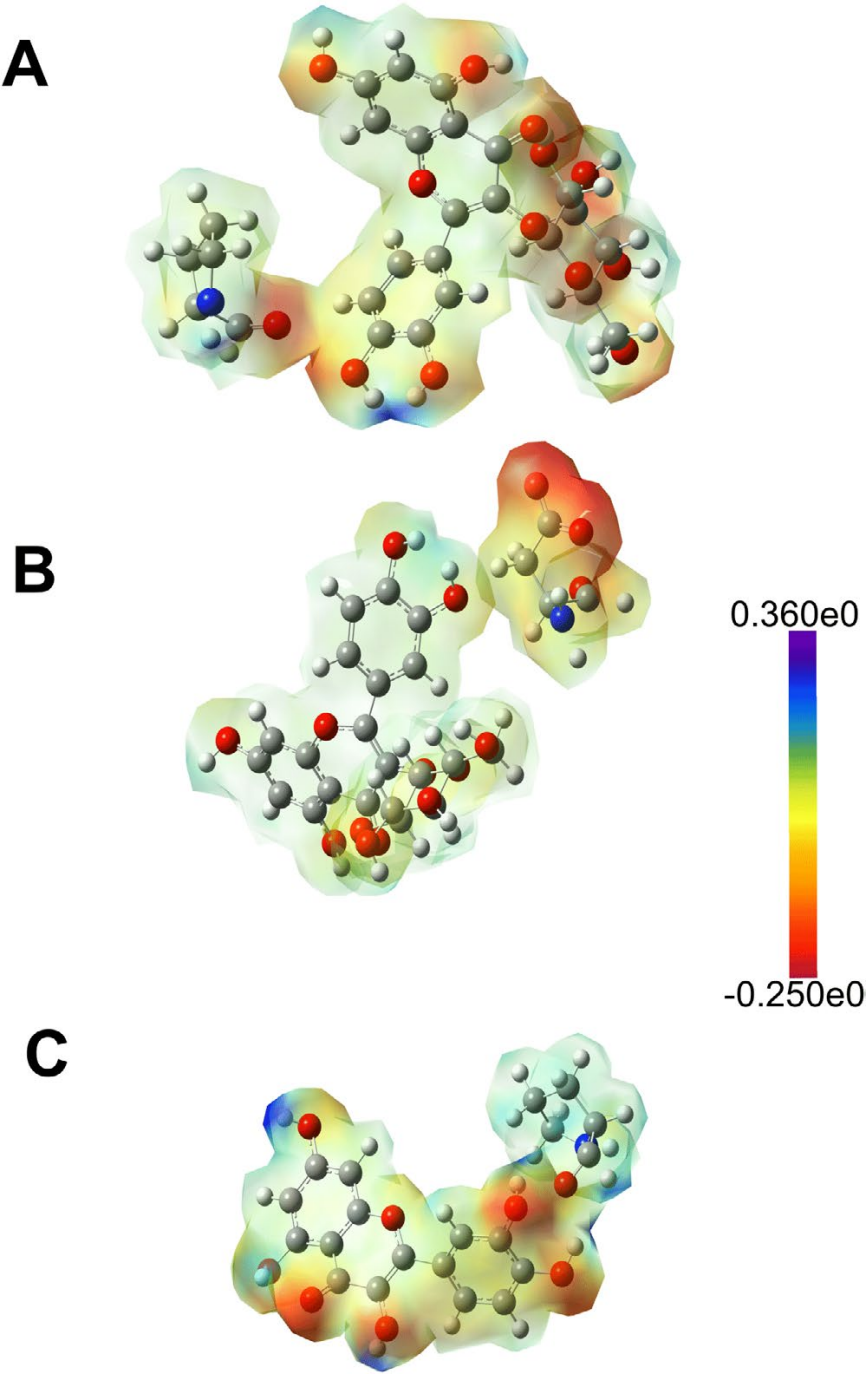
The electrostatic potential isosurface and the projected electron density of the interaction between the Pro62 residue with QUE (A) and ISO (C), in addition to the Asp116 (B) residue with the ISO ligand.

The flavonoid compounds QUE and ISO showed significant repulsive energies with the amino acid Pro62 and, in particular, ISO also denoted repulsive energy with the residue Asp116. Figure 9A–C shows the electrostatic density map of the repulsive interactions involving the amino acid Pro62 with QUE and ISO and the residue Asp116 specifically with ISO.

QUE and ISO, especially the region near the diphenol ring, have a high electron density due to the presence of hydroxyl groups (—OH), which form a repulsive potential when interacting with other regions of similar electron density. On the other hand, the amino acid PRO62 has a specific electron density, especially in the vicinity of the carbonyl group (C=O), making this region partially negative. It should also be taken into account that since these flavonoids contain bulky and rigid groups (aromatic rings with hydroxyl groups), they may have difficulties in accommodation if they are damaged in the vicinity of proline. Since proline is a cyclic amino acid with a rigid conformation, it can also be difficult for other molecules to approach, especially in areas of high electron density, such as carbonyl. The observed repulsive interaction between isoquercetin (near the diphenol ring) and aspartic acid (Asp116) can be explained in a similar way; this repulsion occurs as a direct consequence of the interaction between negative charges, especially near the carboxylic group of Asp116 and the diphenol ring of ISO. These repulsive interactions can modulate the ligand's orientation within the binding site, potentially altering affinity and activation potential. Thus, these interactions allow us to understand the structural constraints and electronic environments of the SIRT6 binding site, highlighting residues that may act as selective determinants for ligand binding. This information can aid in the rationalization of some ligands with lower interaction energies and guide the design of molecules with optimized interactions that minimize unfavorable contacts.

## 4 Conclusions

The characterization of interactions between the SIRT6 protein and the four ligands studied, quercetin (QUE), isoquercetin (ISO), catechin gallate (CG), and trichostatin A (TSA), provides a comprehensive perspective on the molecular dynamics associated with the therapeutic potential of these molecules. Our analysis revealed a hierarchy of binding affinities, with CG showing the strongest interaction, followed by TSA, ISO, and finally, QUE.

Assessment of molecular interactions in different regions of the SIRT6 protein revealed the importance of specific amino acids, such as VAL70, PHE64, PHE82, and PHE86, which play a crucial role in binding site formation. Moreover, residues like PRO62, MET136, MET157, and VAL115 were identified as necessary for positive interactions with ligands.

These findings have significant implications for the development of new therapies for various diseases, including cancer, diabetes, inflammation, and neurodegenerative diseases. A detailed understanding of the molecular interactions between SIRT6 and its ligands may provide valuable insights for the rational development of more effective and selective therapeutic compounds. Furthermore, our computational methodology offers a powerful approach to predict and optimize interactions between proteins and ligands, providing a solid foundation for future drug discovery studies.

In summary, this study provides an understanding of the molecular mechanisms underlying the therapeutic potential of compounds that target the SIRT6 protein, highlighting its importance as a pharmacological target in various health conditions. These discoveries open new perspectives for developing more effective and personalized therapies for metabolic diseases, cancer, and other age‐related conditions.

## Author Contributions

**Érika Geicianny de Carvalho Matias:** methodology, formal analysis, writing – original draft; **Katyanna Sales Bezerra:** methodology, data curation, writing – original draft; **Washington Sales Clemente Junior:** validation, writing – original draft; **Jonas Ivan Nobre Oliveira:** validation, writing – original draft; **Douglas Soares Galvão:** formal analysis, writing – original draft; **Umberto Laino Fulco:** conceptualization, supervision, formal analysis, writing – original draft.

## Funding

This work was supported by Conselho Nacional de Desenvolvimento Científico e Tecnológico; Coordenação de Aperfeiçoamento de Pessoal de Nível Superior.

## Ethics Statement

The authors have nothing to report.

## Consent

The authors have nothing to report.

## Conflicts of Interest

The authors declare no conflicts of interest.

## Supporting information


**Table S1:** Description of SIRT6 residues interacting with the quercetin activator (QUE) identified in the radius of the binding pocket ranging from 2.0 to 10.0 Å. We also expose the regions and groups where there is interaction between SIRT6 residues and the energetic values (in kcal/mol) for *ε* = 10 and *ε* = 40 calculated by the B97D functional combined with the base set 6‐311+G(d,p).
**Table S2:** Description of SIRT6 residues interacting with the isoquercetin activator (ISO) identified in the radius of the binding pocket ranging from 2.0 to 10.0 Å. We also expose the regions and groups where there is interaction between SIRT6 residues and the energetic values (in kcal/mol) for *ε* = 10 and *ε* = 40 calculated by the B97D functional combined with the base set 6‐311+G(d,p).
**Table S3:** Description of SIRT6 residues interacting with the catechin gallate inhibitor (CG) identified in the radius of the binding pocket ranging from 2.0 to 10.0 Å. We also expose the regions and groups where there is interaction between SIRT6 residues and the energetic values (in kcal/mol) for *ε* = 10 and *ε* = 40 calculated by the B97D functional combined with the base set 6‐311+G(d,p).
**Table S4:** Description of SIRT6 residues interacting with the trichostantin A inhibitor (TSA) identified in the radius of the binding pocket ranging from 2.0 to 10.0 Å. We also expose the regions and groups where there is interaction between SIRT6 residues and the energetic values (in kcal/mol) for *ε* = 10 and *ε* = 40 calculated by the B97D functional combined with the base set 6‐311+G(d,p).

## Data Availability

The data that support the findings of this study are openly available in Zenodo at https://zenodo.org/records/17369830.
