# Supplementary material for: Therapeutic Perspectives of SIRT6 Regulation: Computational Analysis of Activation and Inhibition by Bioactive Molecules
Source: J Mol Recognit. 2025 Dec 8;39(1):e70016. doi: 10.1002/jmr.70016 (PMC12686607; doi:10.1002/jmr.70016)
Supplement: Supplementary file 1 — Table S1: Description of SIRT6 residues interacting with the quercetin activator (QUE) identified in the radius of the binding pocket ranging from 2.0 to 10.0 Å. We also expose the regions and groups where there is interaction between SIRT6 residues and the energetic values (in kcal/mol) for ε = 10 and ε = 40 calculated by the B97D functional combined with the base set 6‐311+G(d,p). Table S2: Description of SIRT6 residues interacting with the isoquercetin activator (ISO) identified in the radius of the binding pocket ranging from 2.0 to 10.0 Å. We also expose the regions and groups where there is interaction between SIRT6 residues and the energetic values (in kcal/mol) for ε = 10 and ε = 40 calculated by the B97D functional combined with the base set 6‐311+G(d,p). Table S3: Description of SIRT6 residues interacting with the catechin gallate inhibitor (CG) identified in the radius of the binding pocket ranging from 2.0 to 10.0 Å. We also expose the regions and groups where there is interaction between SIRT6 residues and the energetic values (in kcal/mol) for ε = 10 and ε = 40 calculated by the B97D functional combined with the base set 6‐311+G(d,p). Table S4: Description of SIRT6 residues interacting with the trichostantin A inhibitor (TSA) identified in the radius of the binding pocket ranging from 2.0 to 10.0 Å. We also expose the regions and groups where there is interaction between SIRT6 residues and the energetic values (in kcal/mol) for ε = 10 and ε = 40 calculated by the B97D functional combined with the base set 6‐311+G(d,p). [file JMR-39-e70016-s001.docx]

Supporting Information for:

Therapeutic Perspectives of SIRT6 Regulation: Computational Analysis of Activation and Inhibition by Bioactive Molecules

Érika Geicianny de Carvalho Matias,^†^ Katyanna Sales Bezerra, ^‡^ Washington Sales Clemente Junior,^†^ Jonas Ivan Nobre Oliveira,^†^ Douglas Soares Galvão,‡ and Umberto Laino Fulco∗ ,†

†*Departamento* *de* *Biofísica* *e* *Farmacologia,* *Universidade* *Federal* *do* *Rio* *Grande* *do* *Norte,* *59072-970,* *Natal-RN,* *Brazil.*

‡ *Applied* *Physics* *Department,* *University* *of* *Campinas,* *130838-59,* *Campinas,* *São* *Paulo,* *Brazil.*

∗E-mail: [umbertofulco@gmail.com](mailto:umbertofulco@gmail.com)

Phone: +-55 (84) 3215-3419, Fax: +-55 (84) 3215-3791

Table S1: Description of SIRT6 residues interacting with the quercetin activator (QUE) identified in the radius of the binding pocket ranging from 2.0 to 10.0Å. We also expose the regions and groups where there is interaction between SIRT6 residues and the energetic values (in kcal/mol) for ε=10 and ε=40 calculated by the B97D functional combined with the base set 6-311+G(d,p)

**SIRT6-QUE** **Complex**

| **Residue** | **Atomic Group** | **Radius (Å)** | **Energy (ε=10)** | **Energy (ε=40)** |
| --- | --- | --- | --- | --- |
| ALA53 | i(O1)C | 2.5 | -2.20 | -1.94 |
| ILE61 | i(N1)H | 2.5 | -3.76 | -3.76 |
| PRO62 | i(C15)H14 | 2.5 | -0.71 | -0.45 |
| VAL70 | i(C15)H14 | 2.5 | -2.68 | -2.62 |
| TRP71 | ii(C16)H | 2.5 | -3.57 | -3.51 |
| PHE82 | i(C9)H | 2.5 | -1.93 | -1.91 |
| PHE86 | i(C9)H | 2.5 | -1.75 | -1.75 |
| ASN114 | i(O1)H | 2.5 | -4.40 | -3.99 |
| LYS15 | ii(N2)H22 | 3 | -1.16 | -1.48 |
| ASP63 | i(C12)H | 3 | -1.39 | -1.13 |
| PHE64 | i(C12) | 3 | -4.03 | -3.76 |
| VAL115 | i(C11)H | 3 | -2.99 | -2.82 |
| ASP116 | i(N1)OH | 3 | 0.98 | 0.80 |
| MET157 | ii(C6)H | 3 | -2.66 | -2.58 |
| SER56 | i(N1)OH | 3.5 | -1.06 | -1.03 |
| TRP188 | ii(N2)H18 | 3.5 | -0.68 | -0.85 |
| GLU74 | ii(N2)H22 | 4 | -1.08 | -0.66 |
| GLY52 | i(N1)OH | 4.5 | -0.20 | -0.14 |
| MET136 | ii(C14)H13 | 4.5 | -0.66 | -0.65 |
| ASP187 | ii(N2)H18 | 4.5 | -0.62 | -0.41 |
| ILE55 | i(N1)OH | 5 | -0.26 | -0.25 |
| PRO80 | ii(C2)H | 5 | -0.33 | -0.34 |
| GLN113 | i(C13)O | 5 | -0.30 | -0.43 |
| ILE185 | ii(C6)H | 5 | -0.46 | -0.40 |
| GLY54 | i(N1)OH | 5.5 | -0.16 | -0.10 |
| GLY69 | i(C15)H14 | 5.5 | -0.15 | -0.15 |
| SER112 | i(N1)OH | 5.5 | -0.12 | -0.08 |
| PRO10 | ii(N2)H17 | 6 | 0.05 | -0.06 |
| THR57 | i(C12)H | 6 | -0.21 | -0.19 |
| SER59 | i(N1)H | 6 | -0.05 | -0.05 |
| LEU118 | i(N1)OH | 6 | -0.14 | -0.10 |
| LEU186 | ii(C5)H | 6 | 0.00 | -0.17 |
| PHE11 | ii(N2)H20 | 6.5 | -0.11 | -0.07 |
| THR51 | i(N1)OH | 6.5 | -0.01 | -0.05 |
| HIS95 | i(N1)OH | 6.5 | -0.07 | -0.12 |
| HIS133 | i(C13)O | 6.5 | -0.64 | -0.45 |
| LYS160 | ii(N2)H18 | 6.5 | 0.15 | 0.00 |
| ALA13 | ii(N2)H18 | 7 | -0.07 | -0.05 |

| GLY60 | i(C15)H16 | 7 | 0.14 | 0.11 |
| --- | --- | --- | --- | --- |
| ARG65 | i(C15)H15 | 7 | -0.63 | -0.30 |
| LYS81 | i(C15)H15 | 7 | 0.03 | -0.06 |
| GLY117 | i(N1)OH | 7 | -0.03 | -0.01 |
| HIS119 | i(N1)O | 7 | -0.03 | -0.05 |
| GLY214 | i(N1)OH | 7 | 0.01 | -0.02 |
| HIS68 | i(C15)H15 | 7.5 | -0.06 | -0.04 |
| GLY158 | ii(C17)H19 | 7.5 | -0.07 | -0.04 |
| THR215 | i(N1)OH | 7.5 | -0.02 | -0.03 |
| ALA58 | i(N1)H | 8 | -0.03 | -0.03 |
| GLY66 | i(C15)H14 | 8 | -0.01 | -0.02 |
| THR84 | i(C15)H16 | 8 | -0.06 | -0.04 |
| ALA89 | i(N1)H | 8 | 0.00 | -0.02 |
| VAL153 | ii(C14)H11 | 8 | -0.06 | -0.04 |
| THR72 | i(C15)H14 | 8.5 | -0.02 | -0.03 |
| MET73 | i(C15)H15 | 8.5 | 0.00 | -0.03 |
| ASP83 | i(C15)H16 | 8.5 | -0.09 | 0.00 |
| THR92 | i(N1)O | 8.5 | 0.01 | -0.01 |
| GLY134 | i(C13)O | 8.5 | 0.06 | -0.01 |
| ASN135 | i(C11)H | 8.5 | -0.04 | -0.05 |
| GLU189 | ii(C17)H18 | 8.5 | -0.14 | -0.06 |
| LEU213 | i(N1)OH | 8.5 | 0.02 | 0.00 |
| THR12 | ii(C16)H20 | 9 | -0.02 | -0.02 |
| ALA79 | ii(C3)H | 9 | 0.01 | -0.02 |
| THR85 | ii(C14)H11 | 9 | -0.05 | -0.03 |
| GLU131 | i(N1)O | 9 | -0.02 | -0.02 |
| PHE137 | i(N1)H | 9 | -0.07 | -0.05 |
| THR156 | ii(C14)H13 | 9 | -0.04 | -0.06 |
| GLU75 | ii(C16)H22 | 9.5 | -0.09 | -0.04 |
| PRO91 | i(N1)O | 9.5 | -0.03 | -0.01 |
| ASP183 | ii(C6)H | 9.5 | 0.03 | 0.00 |
| ASP190 | ii(C17)H18 | 9.5 | 0.05 | 0.00 |
| SER216 | i(N1)OH | 9.5 | -0.02 | -0.02 |
| ILE219 | i(C13)O | 9.5 | -0.03 | -0.03 |
| CYS18 | ii(C16)H22 | 10 | 0.01 | -0.01 |
| HIS50 | i(N1)OH | 10 | -0.07 | -0.03 |
| LEU78 | ii(C3)H | 10 | -0.08 | -0.06 |
| ARG90 | i(N1)H | 10 | -0.05 | -0.02 |
| VAL111 | i(N1)OH | 10 | -0.04 | -0.02 |
| VAL120 | i(N1)O | 10 | -0.06 | -0.02 |
| LEU129 | i(N1)OH | 10 | -0.04 | -0.02 |
| LEU132 | i(C13)O | 10 | -0.10 | -0.04 |
| GLY155 | ii(C14)H13 | 10 | -0.09 | -0.05 |
| LEU159 | ii(C5)H | 10 | -0.03 | -0.02 |
| THR184 | ii(C6)H | 10 | 0.01 | -0.02 |
| VAL258 | i(N1)OH | 10 | -0.01 | -0.01 |

Table S2: Description of SIRT6 residues interacting with the isoquercetin activator (ISO) identified in the radius of the binding pocket ranging from 2.0 to 10.0Å. We also expose the regions and groups where there is interaction between SIRT6 residues and the energetic values (in kcal/mol) for ε=10 and ε=40 calculated by the B97D functional combined with the base set 6-311+G(d,p)

**SIRT6-ISO** **Complex**

| **Residue** | **Atomic Group** | **Radius (Å)** | **Energy (ε=10)** | **Energy (ε=40)** |
| --- | --- | --- | --- | --- |
| PHE64 | iii(C5)H | 2 | -5.43 | -5.36 |
| ALA53 | iii(C20)OH | 2.5 | -0.60 | -0.64 |
| PRO62 | i(C19)H | 2.5 | 2.63 | 2.87 |
| VAL70 | i(C18)H | 2.5 | -2.95 | -2.95 |
| TRP71 | iii(C3)OH | 2.5 | -2.66 | -2.53 |
| PHE82 | ii(C12)H | 2.5 | -1.65 | -1.61 |
| VAL115 | iii(C6)OH | 2.5 | -3.41 | -3.31 |
| HIS133 | iii(C6)H | 2.5 | -2.34 | -2.31 |
| ILE61 | i(C20)OH | 3 | -1.55 | -1.55 |
| ASP63 | i(C19)H | 3 | -1.29 | -0.86 |
| PRO80 | ii(C11)OH | 3 | -1.46 | -1.44 |
| ASP116 | i(C21)OH | 3 | 2.49 | 2.81 |
| MET157 | iii(O2)H | 3 | -0.97 | -1.01 |
| PHE86 | i(C16)H | 3.5 | -3.42 | -3.41 |
| GLN113 | iii(C6)H | 4 | -0.43 | -0.47 |
| MET136 | iii(O1)C | 4 | -1.81 | -1.75 |
| ILE185 | i(C6)H | 4 | -1.66 | -1.68 |
| LEU186 | iii(C4)H | 4 | -0.92 | -0.69 |
| ASN114 | i(C21)OH | 4.5 | -1.17 | -1.08 |
| TRP188 | iii(C3)OH | 4.5 | -1.06 | -1.04 |
| SER56 | i(C20)OH | 5 | -0.20 | -0.25 |
| ARG65 | iii(C6)OH | 5 | -0.18 | -0.25 |
| ASP187 | iii(C4)OH | 5 | -0.47 | -0.36 |
| THR57 | i(C20)OH | 5.5 | -0.12 | -0.16 |
| ILE219 | iii(C6)OH | 5.5 | -0.19 | -0.18 |
| GLY52 | i(C20)OH | 6 | 0.16 | 0.05 |
| GLY54 | i(C20)OH | 6 | -0.06 | -0.06 |
| GLY69 | i(C19)H | 6 | 0.00 | -0.04 |
| ALA79 | ii(C11)OH | 6 | -0.07 | -0.11 |
| GLY134 | iii(C6)H | 6 | -0.11 | -0.10 |
| LYS15 | iii(C3)OH | 6.5 | -0.36 | -0.18 |
| LYS81 | ii(C11)OH | 6.5 | -0.39 | -0.34 |
| GLY117 | i(C21)OH | 6.5 | -0.07 | -0.08 |
| LEU118 | i(C21)OH | 6.5 | -0.07 | -0.08 |
| ILE55 | i(C20)OH | 7 | -0.03 | -0.05 |
| LEU132 | iii(C6)H | 7 | -0.11 | -0.10 |
| GLY158 | iii(C4)H | 7 | -0.01 | -0.02 |

| THR184 | iii(C6)H | 7 | -0.10 | -0.09 |
| --- | --- | --- | --- | --- |
| SER59 | i(C20)OH | 7.5 | -0.01 | -0.03 |
| GLY66 | i(C19)H | 7.5 | -0.01 | -0.02 |
| HIS68 | i(C19)H | 7.5 | -0.02 | -0.04 |
| ASN135 | iii(C6)H | 7.5 | -0.15 | -0.13 |
| LEU192 | iii(C6)OH | 7.5 | -0.05 | -0.04 |
| PRO10 | iii(C3)OH | 8 | -0.10 | -0.05 |
| ALA13 | iii(C3)OH | 8 | -0.03 | -0.03 |
| GLY60 | i(C20)OH | 8 | -0.12 | -0.13 |
| MET73 | ii(C12)H | 8 | -0.03 | -0.05 |
| GLU74 | iii(C3)OH | 8 | 0.09 | -0.03 |
| SER112 | i(C21)OH | 8 | 0.00 | -0.03 |
| VAL153 | ii(C15)OH | 8 | -0.10 | -0.08 |
| GLU189 | iii(C4)OH | 8 | 0.02 | -0.02 |
| ASP190 | iii(C4)OH | 8 | -0.01 | -0.02 |
| GLY214 | i(C20)OH | 8 | -0.08 | -0.03 |
| SER222 | iii(C5)OH | 8 | -0.02 | -0.02 |
| THR72 | i(C18)H | 8.5 | -0.01 | -0.04 |
| THR84 | ii(C11)OH | 8.5 | -0.05 | -0.05 |
| THR85 | ii(C10)H | 8.5 | -0.10 | -0.07 |
| HIS119 | i(C21)OH | 8.5 | -0.07 | -0.04 |
| GLU131 | iii(C6)H | 8.5 | -0.04 | -0.05 |
| PHE137 | i(C16)H | 8.5 | -0.06 | -0.07 |
| GLY155 | ii(C15)OH | 8.5 | -0.01 | -0.02 |
| THR156 | ii(C9)O | 8.5 | -0.13 | -0.15 |
| LEU159 | iii(C6)H | 8.5 | -0.04 | -0.04 |
| THR51 | i(C21)OH | 9 | 0.04 | -0.01 |
| ALA58 | i(C20)OH | 9 | -0.02 | -0.02 |
| LEU78 | ii(C11)OH | 9 | -0.01 | -0.02 |
| ASP83 | ii(C11)OH | 9 | 0.00 | 0.02 |
| ALA89 | i(C21)OH | 9 | -0.02 | -0.02 |
| HIS95 | i(C20)OH | 9 | -0.03 | -0.02 |
| VAL154 | ii(C15)OH | 9 | -0.02 | -0.04 |
| ASP183 | iii(C2)H | 9 | 0.05 | -0.01 |
| PRO193 | iii(C6)H | 9 | -0.01 | -0.01 |
| THR215 | i(C20)OH | 9 | -0.03 | -0.02 |
| ASP14 | iii(C4)OH | 9.5 | 0.06 | 0.00 |
| SER216 | iii(C6)OH | 9.5 | 0.00 | -0.01 |
| PHE11 | iii(C3)OH | 10 | 0.01 | -0.01 |
| GLU87 | ii(C15)OH | 10 | 0.17 | 0.04 |
| THR92 | i(C20)OH | 10 | 0.00 | -0.01 |
| LYS160 | iii(C2)H | 10 | -0.08 | -0.04 |
| GLN218 | iii(C6)OH | 10 | -0.02 | -0.03 |
| ARG220 | iii(C6)OH | 10 | -0.01 | -0.01 |
| PRO221 | iii(C6)OH | 10 | -0.01 | -0.01 |
| GLY223 | iii(C6)OH | 10 | -0.01 | -0.01 |

Table S3: Description of SIRT6 residues interacting with the catechin gallate inhibitor (CG) identified in the radius of the binding pocket ranging from 2.0 to 10.0Å. We also expose the regions and groups where there is interaction between SIRT6 residues and the energetic values (in kcal/mol) for ε=10 and ε=40 calculated by the B97D functional combined with the base set 6-311+G(d,p)

**SIRT6-CG** **Complex**

| **Residue** | **Atomic Group** | **Radius (Å)** | **Energy (ε=10)** | **Energy (ε=40)** |
| --- | --- | --- | --- | --- |
| PRO62 | iii(C3)OH | 2 | -4.22 | -3.96 |
| THR156 | i(C21)OH | 2 | -6.24 | -5.71 |
| VAL70 | iii(C2)H | 2.5 | -3.62 | -3.55 |
| TRP71 | ii(C11)OH | 2.5 | -1.70 | -1.60 |
| VAL115 | iii(C5)H | 2.5 | -2.38 | -2.29 |
| ASP116 | iii(C4)OH | 2.5 | -1.39 | -1.39 |
| MET136 | i(C22)H | 2.5 | -5.83 | -5.68 |
| GLY155 | i(C20)OH | 2.5 | -1.66 | -1.66 |
| MET157 | i(C22)H | 2.5 | -7.63 | -7.42 |
| ILE185 | i(C22)H | 2.5 | -2.85 | -2.80 |
| ALA53 | i(C3)OH | 3 | -0.90 | -0.75 |
| ILE61 | i(C4)OH | 3 | -1.96 | -2.07 |
| ASP63 | iii(C3)OH | 3 | -2.57 | -2.28 |
| PHE82 | ii(C7)H | 3 | -2.73 | -2.67 |
| PHE86 | ii(C7)H | 3 | -3.97 | -4.01 |
| VAL154 | i(C21)OH | 3 | -0.79 | -0.80 |
| PHE64 | iii(C6) | 3.5 | -4.59 | -4.44 |
| VAL153 | i(C20)OH | 3.5 | -1.55 | -1.63 |
| ASP183 | i(C21)OH | 3.5 | -1.06 | -0.75 |
| PRO80 | ii(C15)OH | 4 | -0.80 | -0.78 |
| ASN114 | iii(C4)OH | 4.5 | -0.57 | -0.54 |
| SER56 | iii(C3)OH | 5 | -0.70 | -0.50 |
| THR57 | iii(C3)OH | 5 | -0.13 | -0.11 |
| GLU74 | ii(C11)OH | 5 | -0.22 | -0.20 |
| THR184 | i(C21)OH | 5 | -0.23 | -0.29 |
| LYS15 | ii(C11)OH | 6 | -0.26 | -0.15 |
| GLY69 | iii(C2)H | 6 | -0.14 | -0.11 |
| GLY117 | iii(C4)OH | 6 | -0.13 | -0.09 |
| ASN135 | i(C21)OH | 6 | -0.54 | -0.51 |
| PHE137 | i(C21)OH | 6 | -0.26 | -0.24 |
| GLY158 | i(C21)OH | 6 | -0.05 | -0.07 |
| THR162 | i(C21)OH | 6 | -0.04 | -0.05 |
| GLY54 | iii(C3)OH | 6.5 | -0.06 | -0.03 |
| LEU118 | iii(C4)OH | 6.5 | -0.14 | -0.10 |
| VAL138 | i(C21)OH | 6.5 | -0.14 | -0.10 |
| LEU159 | i(C21)OH | 6.5 | 0.01 | -0.03 |
| ARG164 | i(C20)OH | 6.5 | 0.16 | 0.01 |

| TRP188 | ii(C12)H | 6.5 | -0.15 | -0.14 |
| --- | --- | --- | --- | --- |
| GLY52 | iii(C3)OH | 7 | -0.27 | -0.17 |
| ARG65 | iii(C2)H | 7 | -0.44 | -0.17 |
| GLN113 | i(C4)OH | 7 | -0.43 | -0.42 |
| LYS160 | i(C21)OH | 7 | -0.09 | -0.06 |
| GLY60 | iii(C3)OH | 7 | 0.06 | 0.04 |
| HIS68 | iii(C2)H | 7.5 | -0.05 | -0.04 |
| ALA79 | ii(C10)H | 7.5 | -0.07 | -0.06 |
| HIS133 | i(C22)H | 7.5 | -0.20 | -0.14 |
| GLY134 | i(C22)H | 7.5 | 0.04 | -0.03 |
| GLU139 | i(C21)OH | 7.5 | -0.25 | -0.09 |
| THR152 | i(C20)OH | 7.5 | -0.04 | -0.08 |
| LEU186 | i(C22)H | 7.5 | -0.08 | -0.15 |
| ASP187 | ii(C12)H | 7.5 | 0.23 | 0.02 |
| ILE55 | iii(C3)OH | 8 | -0.09 | -0.07 |
| SER59 | iii(C4)OH | 8 | 0.00 | -0.02 |
| GLY66 | iii(C2)H | 8 | 0.00 | -0.01 |
| LYS81 | ii(C15)OH | 8 | 0.06 | -0.04 |
| GLU87 | i(C19)OH | 8 | -0.40 | -0.15 |
| ALA89 | iii(C4)OH | 8 | -0.01 | -0.02 |
| ARG182 | i(C21)OH | 8 | 0.14 | 0.03 |
| THR72 | iii(C2)H | 8.5 | 0.00 | 0.00 |
| MET73 | ii(C10)H | 8.5 | -0.03 | -0.04 |
| THR84 | iii(C4)OH | 8.5 | -0.05 | -0.07 |
| THR85 | i(C19)OH | 8.5 | 0.01 | -0.04 |
| SER112 | iii(C4)OH | 8.5 | -0.06 | -0.03 |
| GLY214 | iii(C3)OH | 8.5 | 0.04 | 0.00 |
| PRO10 | ii(C11)OH | 9 | -0.16 | -0.16 |
| PHE11 | ii(C11)OH | 9 | -0.01 | -0.01 |
| ALA13 | ii(C11)OH | 9 | -0.02 | -0.02 |
| LEU78 | ii(C10)H | 9 | -0.02 | -0.02 |
| HIS95 | iii(C4)OH | 9 | -0.03 | -0.02 |
| HIS119 | iii(C4)OH | 9 | 0.01 | -0.02 |
| ARG150 | i(C20)OH | 9 | 0.12 | 0.01 |
| ALA58 | iii(C3)OH | 9.5 | 0.00 | -0.01 |
| GLU75 | ii(C11)OH | 9.5 | 0.05 | 0.01 |
| ASP83 | iii(C3)OH | 9.5 | -0.12 | -0.03 |
| GLU131 | iii(C4)OH | 9.5 | -0.02 | -0.02 |
| GLU140 | i(C21)OH | 9.5 | -0.05 | -0.02 |
| ALA161 | i(C21)OH | 9.5 | -0.02 | -0.01 |
| THR215 | iii(C3)OH | 9.5 | -0.02 | -0.01 |
| SER216 | iii(C3)OH | 9.5 | 0.00 | -0.01 |
| THR51 | iii(C3)OH | 10 | -0.03 | -0.02 |
| ARG121 | iii(C4)OH | 10 | 0.11 | 0.01 |

Table S4: Description of SIRT6 residues interacting with the trichostantin A inhibitor (TSA) identified in the radius of the binding pocket ranging from 2.0 to 10.0Å. We also expose the regions and groups where there is interaction between SIRT6 residues and the energetic values (in kcal/mol) for ε=10 and ε=40 calculated by the B97D functional combined with the base set 6-311+G(d,p)

**SIRT6-TSA** **Complex**

| **Residue** | **Atomic Group** | **Radius (Å)** | **Energy (ε=10)** | **Energy (ε=40)** |
| --- | --- | --- | --- | --- |
| ALA53 | i(O1)C | 2.5 | -2.20 | -1.94 |
| ILE61 | i(N1)H | 2.5 | -3.76 | -3.76 |
| PRO62 | i(C15)H14 | 2.5 | -0.71 | -0.45 |
| VAL70 | i(C15)H14 | 2.5 | -2.68 | -2.62 |
| TRP71 | ii(C16)H | 2.5 | -3.57 | -3.51 |
| PHE82 | i(C9)H | 2.5 | -1.93 | -1.91 |
| PHE86 | i(C9)H | 2.5 | -1.75 | -1.75 |
| ASN114 | i(O1)H | 2.5 | -4.40 | -3.99 |
| LYS15 | ii(N2)H22 | 3 | -1.16 | -1.48 |
| ASP63 | i(C12)H | 3 | -1.39 | -1.13 |
| PHE64 | i(C12) | 3 | -4.03 | -3.76 |
| VAL115 | i(C11)H | 3 | -2.99 | -2.82 |
| ASP116 | i(N1)OH | 3 | 0.98 | 0.80 |
| MET157 | ii(C6)H | 3 | -2.66 | -2.58 |
| SER56 | i(N1)OH | 3.5 | -1.06 | -1.03 |
| TRP188 | ii(N2)H18 | 3.5 | -0.68 | -0.85 |
| GLU74 | ii(N2)H22 | 4 | -1.08 | -0.66 |
| GLY52 | i(N1)OH | 4.5 | -0.20 | -0.14 |
| MET136 | ii(C14)H13 | 4.5 | -0.66 | -0.65 |
| ASP187 | ii(N2)H18 | 4.5 | -0.62 | -0.41 |
| ILE55 | i(N1)OH | 5 | -0.26 | -0.25 |
| PRO80 | ii(C2)H | 5 | -0.33 | -0.34 |
| GLN113 | i(C13)O | 5 | -0.30 | -0.43 |
| ILE185 | ii(C6)H | 5 | -0.46 | -0.40 |
| GLY54 | i(N1)OH | 5.5 | -0.16 | -0.10 |
| GLY69 | i(C15)H14 | 5.5 | -0.15 | -0.15 |
| SER112 | i(N1)OH | 5.5 | -0.12 | -0.08 |
| PRO10 | ii(N2)H17 | 6 | 0.05 | -0.06 |
| THR57 | i(C12)H | 6 | -0.21 | -0.19 |
| SER59 | i(N1)H | 6 | -0.05 | -0.05 |
| LEU118 | i(N1)OH | 6 | -0.14 | -0.10 |
| LEU186 | ii(C5)H | 6 | 0.00 | -0.17 |
| PHE11 | ii(N2)H20 | 6.5 | -0.11 | -0.07 |
| THR51 | i(N1)OH | 6.5 | -0.01 | -0.05 |
| HIS95 | i(N1)OH | 6.5 | -0.07 | -0.12 |
| HIS133 | i(C13)O | 6.5 | -0.64 | -0.45 |
| LYS160 | ii(N2)H18 | 6.5 | 0.15 | 0.00 |

| ALA13 | ii(N2)H18 | 7 | -0.07 | -0.05 |
| --- | --- | --- | --- | --- |
| GLY60 | i(C15)H16 | 7 | 0.14 | 0.11 |
| ARG65 | i(C15)H15 | 7 | -0.63 | -0.30 |
| LYS81 | i(C15)H15 | 7 | 0.03 | -0.06 |
| GLY117 | i(N1)OH | 7 | -0.03 | -0.01 |
| HIS119 | i(N1)O | 7 | -0.03 | -0.05 |
| GLY214 | i(N1)OH | 7 | 0.01 | -0.02 |
| HIS68 | i(C15)H15 | 7.5 | -0.06 | -0.04 |
| GLY158 | ii(C17)H19 | 7.5 | -0.07 | -0.04 |
| THR215 | i(N1)OH | 7.5 | -0.02 | -0.03 |
| ALA58 | i(N1)H | 8 | -0.03 | -0.03 |
| GLY66 | i(C15)H14 | 8 | -0.01 | -0.02 |
| THR84 | i(C15)H16 | 8 | -0.06 | -0.04 |
| ALA89 | i(N1)H | 8 | 0.00 | -0.02 |
| VAL153 | ii(C14)H11 | 8 | -0.06 | -0.04 |
| THR72 | i(C15)H14 | 8.5 | -0.02 | -0.03 |
| MET73 | i(C15)H15 | 8.5 | 0.00 | -0.03 |
| ASP83 | i(C15)H16 | 8.5 | -0.09 | 0.00 |
| THR92 | i(N1)O | 8.5 | 0.01 | -0.01 |
| GLY134 | i(C13)O | 8.5 | 0.06 | -0.01 |
| ASN135 | i(C11)H | 8.5 | -0.04 | -0.05 |
| GLU189 | ii(C17)H18 | 8.5 | -0.14 | -0.06 |
| LEU213 | i(N1)OH | 8.5 | 0.02 | 0.00 |
| THR12 | ii(C16)H20 | 9 | -0.02 | -0.02 |
| ALA79 | ii(C3)H | 9 | 0.01 | -0.02 |
| THR85 | ii(C14)H11 | 9 | -0.05 | -0.03 |
| GLU131 | i(N1)O | 9 | -0.02 | -0.02 |
| PHE137 | i(N1)H | 9 | -0.07 | -0.05 |
| THR156 | ii(C14)H13 | 9 | -0.04 | -0.06 |
| GLU75 | ii(C16)H22 | 9.5 | -0.09 | -0.04 |
| PRO91 | i(N1)O | 9.5 | -0.03 | -0.01 |
| ASP183 | ii(C6)H | 9.5 | 0.03 | 0.00 |
| ASP190 | ii(C17)H18 | 9.5 | 0.05 | 0.00 |
| SER216 | i(N1)OH | 9.5 | -0.02 | -0.02 |
| ILE219 | i(C13)O | 9.5 | -0.03 | -0.03 |
| CYS18 | ii(C16)H22 | 10 | 0.01 | -0.01 |
| HIS50 | i(N1)OH | 10 | -0.07 | -0.03 |
| LEU78 | ii(C3)H | 10 | -0.08 | -0.06 |
| ARG90 | i(N1)H | 10 | -0.05 | -0.02 |
| VAL111 | i(N1)OH | 10 | -0.04 | -0.02 |
| VAL120 | i(N1)O | 10 | -0.06 | -0.02 |
| LEU129 | i(N1)OH | 10 | -0.04 | -0.02 |
| LEU132 | i(C13)O | 10 | -0.10 | -0.04 |
| GLY155 | ii(C14)H13 | 10 | -0.09 | -0.05 |
| LEU159 | ii(C5)H | 10 | -0.03 | -0.02 |
| THR184 | ii(C6)H | 10 | 0.01 | -0.02 |
| VAL258 | i(N1)OH | 10 | -0.01 | -0.01 |
